# Tracing metal sources and groundwater flow paths in the Upper Animas River watershed using rare earth elements and stable isotopes

**DOI:** 10.1144/geochem2024-023

**Published:** 2025-02

**Authors:** Connor P. Newman, Rory Cowie, Richard T. Wilkin, Alexis Navarre-Sitchler

**Affiliations:** 1 U.S. Geological Survey, Colorado Water Science Center, Denver, CO, USA; 2 Colorado School of Mines, Geochemistry Program, Department of Geology and Geological Engineering, Golden, CO, USA; 3 Alpine Water Resources LLC, Silverton, CO, USA; 4 U.S. Environmental Protection Agency, Office of Research and Development, Groundwater Characterization and Remediation Division, OK, USA

**Keywords:** acid mine drainage, alteration assemblages, hydraulic bulkheads, mine workings, Colorado

## Abstract

Groundwater flow paths and processes that govern metal mobility and transport are difficult to characterize in mountainous bedrock watersheds. Despite the difficulty in holistic characterization, conceptual understanding of subsurface hydrologic and geochemical processes is key to developing remediation plans for locations affected by acid mine drainage, such as the Upper Animas River watershed in southwestern Colorado, USA. Stable isotopes of water and rare earth elements were utilized to evaluate groundwater flow and metal sources within this complex catchment. Stable isotope samples collected from draining mine adits and springs display systematic spatial variation wherein sample sites at higher elevations have greater seasonal variability than sites at lower elevations. The Upper Cement Creek watershed, where multiple draining mines are present, displays the lowest seasonal variation in stable isotopic signatures, potentially indicating the presence of a large, well-mixed volume of groundwater storage or interbasin groundwater flow. Rare earth elements display statistically significant variation between different alteration styles in the catchment. Overprinting of regional propylitic alteration is evident based on enrichment of middle rare earth elements in acidic springs and mines that are not spatially associated with surficial exposures of acid generating alteration styles. Europium anomaly and middle rare earth enrichment signatures from two flooded mine tunnels on opposite sides of a watershed divide indicate connections to the same subsurface flooded mine workings.

Sources of metals in watersheds affected by acid mine drainage (AMD) commonly include mine waste piles, draining mine adits and natural sulfide weathering (Wolkersdorfer et al. 2020). Remediation of high-metal, low-pH waters is often complicated by mixing of fluids from these various sources and uncertainty in the geochemical and hydrologic processes that transport metals to streams (Williams et al. 2015; Manning et al. 2022; Runkel et al. 2023). As a result of uncertainty in the source of metals and processes that regulate metal fluxes, remediation efforts may be ineffective in some instances (Runkel et al. 2016; Duraz et al. 2023). Common factors that hinder remediation include mixing of metals from multiple spatially distinct sources and subsequent difficulty in separating mining-influenced water quality from water quality affected by natural weathering of sulfides (Nordstrom 2015). Appropriate definition of spatially variable metal sources, and the processes that affect metal attenuation and transport, could therefore be beneficial for robust remediation planning.

The locations of AMD generating sources, the processes that generate AMD, and transport of metals in groundwater and stream systems all determine the impact of AMD on downstream water quality. Because the inferences that can be made about AMD generation and transport are dependent on the methods used, discussion of the variety of techniques is beneficial. Methods that are commonly used to investigate metal sources and governing processes in AMD systems include evaluation of both surface water and groundwater. Tracer-injection and breakthrough analysis in streams are used to identify locations of relatively high influx of AMD to streams, including specific stream reaches where mining inflows are highest (Runkel et al. 2023). Tracer-injection may be combined with water level measurements from near- and in-stream piezometers to quantify fluxes of metals from subsurface sources v. surface sources such as mine waste piles (Manning et al. 2022). Both natural tracers (such as stable isotopes of water: ${\text{δ}}^{2}\text{H}$ and ${\text{δ}}^{18}\text{O}$) and introduced tracers (such as fluorescent dyes or conservative ions) may be used to directly identify flow paths of water within mine workings and fractured bedrock (Cowie et al. 2014; Wolkersdorfer et al. 2020; Langman et al. 2023). Metal geochemical signatures, such as patterns in rare earth element (REE) distributions, may be useful for understanding longitudinal transport of solutes and processes leading to solute mobilization (Leybourne et al. 1998; Verplanck et al. 1999; Göb et al. 2013; Olías et al. 2018). Geochemical behaviour of REEs is also useful for identifying groundwater recharge zones and mixing along flow paths (Johannesson et al. 1997; Tweed et al. 2006; Duvert et al. 2015). When used alone or in conjunction with one another, this variety of approaches can provide insight into the sources of metals and processes that control metal distributions along a contaminant transport pathway.

This study tests the use of REEs and stable isotopes of water in understanding mobilization and transport of metals through the subsurface and to streams in an AMD-affected watershed in southwestern Colorado, the Upper Animas River watershed. The results provide regional context for metal loading in the headwaters of the San Juan River by describing processes that mobilize solutes, which could be evaluated in other areas of the watershed where similar environmental concerns exist. Results also provide process-based information on the application of these tracers in other diverse hydrologic and geochemical environments affected by AMD.

## Study area description

**Fig. 1.**
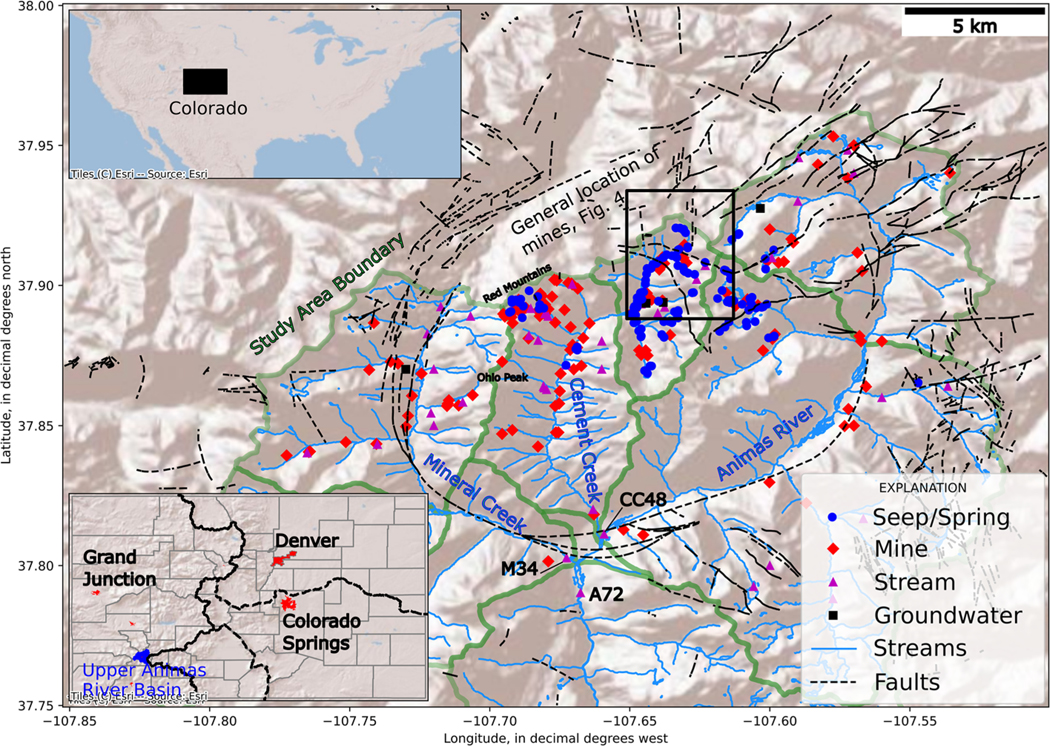
Study area map and data collection sites. The upper-left inset map indicates the location of Colorado within the United States. The lower-left inset map shows the location of the Upper Animas River watershed within Colorado, with primary population centres shown in red.

The Upper Animas River watershed is a high elevation region of southwestern Colorado, USA, that forms one of the headwaters of the San Juan River (Fig. 1). The area is highly mineralized and represents one of the most historically productive mining regions of Colorado, where mining primarily occurred between the 1870s and 1990s (Jones 2007). The effects of mining on watershed health, aquatic ecosystems, aqueous geochemistry and physical hydrology were evaluated in an extensive study in the late 1990s (Church et al. 2007). Although that study characterized much of the mining-related contamination at the time, subsequent remediation measures included the installation and closure of hydraulic bulkheads within draining mine adits. The closure of adits and impoundment of water within open mine workings has modified the geochemistry and hydrology of the watershed (Walton-Day et al. 2021). Prior to bulkhead installation, the American Tunnel (an approximately 3.2 km long haulage and drainage tunnel that crosses beneath a watershed divide; Jones 2007) was the primary source of mine-affected discharge within the watershed. Beginning in 1996 and ending in 2003, three bulkheads were installed within the American Tunnel (sequentially through time, AT #1, AT #2, AT #3) and one bulkhead was installed in the Mogul mine, which was connected via underground workings to the Sunnyside mine and the American Tunnel. Additional bulkheads were installed elsewhere throughout the watershed (Walton-Day et al. 2021). Bulkhead installation rerouted flow within interconnected mine workings, and potentially along naturally occurring fractures, to mines that were previously dry, leading to measurable discharge from a greater number of mine adits and increasing the distribution of AMD sources within the watershed (Walton-Day et al. 2021). Bulkhead installation also resulted in the flooding of the American Tunnel, Sunnyside mine and Mogul mine.

The Upper Animas River watershed is also the location of the Gold King mine, where an uncontrolled release of AMD occurred in 2015. This release resulted in the discharge of approximately 11 300 cubic metres of acidic and metal-rich water into North Fork Cement Creek, which ultimately was transported to the Animas River, San Juan River and Colorado River (Rodriguez-Freire et al. 2016). Subsequent to the Gold King mine release, a subset of the mines within the Upper Animas River watershed were classified as the Bonita Peak Mining District Superfund Site by the US Environmental Protection Agency (EPA; US Environmental Protection Agency 2024).

The study area is intensely mineralized and contains quartz–sericite–pyrite (QSP), vein-related QSP (VQSP), acid sulfate (AS), weak sericite–pyrite (WSP), propylitic (PROP) and combined weak sericite–pyrite/propylitic (WSP–PROP) alteration styles, as well as quaternary undifferentiated (QU) surficial material. These alteration styles display unique solid and aqueous geochemical signatures; QSP and AS typically display the highest metal concentrations whereas PROP displays the lowest metal concentrations. Both QSP and AS alteration are most abundant on the Red Mountains and Ohio Peak (Fig. 1), whereas the majority of the remainder of the study area is characterized by regional PROP and localized areas of VQSP and WSP (Bove et al. 2007).

In a regional analysis using water-quality data from watershed outlets and remote sensing estimates of surficial alteration styles, Yager et al. (2013) identified that watersheds with greater proportions of QSP and AS displayed more acidic stream pH at their outlets. Contrastingly, watersheds with a greater proportion of PROP alteration were generally characterized by circumneutral stream pH at their outlets. A third group of watersheds was identified however, which were dominated by PROP alteration but also displayed acidic streams. This third grouping of PROP-dominated yet acidic streams indicated that surficial alteration is a potentially promising method for identifying spatially distinct metal sources, but that the approach was not universally effective. Watersheds with substantial PROP alteration mapped at the surface, but which paradoxically have acid streams, may be caused by anthropogenic modifications or because the surficial alteration assemblage is not representative of the bulk material in the watershed that may affect water quality. Anthropogenic modifications could be geochemical such as increasing the surface area and reactivity of sulfide minerals, or hydrologic wherein groundwater that previously flowed to a different watershed has been rerouted.

Several outstanding questions related to groundwater flow paths and the sources and transport of metals in the Upper Animas River watershed form the impetus for this study. First, given the substantial modifications to the hydrology of the interconnected system of mine working induced by bulkheading (Walton-Day et al. 2021), to what degree are groundwater emergence points (springs and draining mine adits) connected across hydrographic basins? Previous analysis prior to the installation of bulkheads indicated that interbasin flow may occur once bulkheads were installed and the system of underground workings were filled (Simon Hydro-Search 1993). This analysis uses stable isotopes of water to evaluate potential interbasin flow because isotopes have been useful in tracing flow paths in other studies of complex mine-affected hydrologic systems (Cowie et al. 2014; Langman et al. 2023). Second, given the variable sources of metals known within the study area (Bove et al. 2007; Nash and Fey 2007; Yager et al. 2013), to what degree are metals derived from spatially distinct sources differentiable? This analysis uses REEs to evaluate metal sourcing given their utility in other areas affected by AMD for identifying metal transport processes (Leybourne et al. 1998, 2000; Pérez-López et al. 2010; Göb et al. 2013; Olías et al. 2018; Liu et al. 2024) and in identifying local to regional groundwater flow paths and hydrologic connectivity (Gosselin et al. 1992; Johannesson et al. 1997; Duvert et al. 2015).

## Methods

Samples used in this analysis were compiled from several existing datasets (Table 1). Following the designation of the Bonita Peak Mining District Superfund Site, numerous springs and draining mines were sampled by the Mountain Studies Institute (MSI) for stable isotopes of water (${\text{δ}}^{2}\text{H}$ and ${\text{δ}}^{18}\text{O}$) to evaluate groundwater connectivity (Cowie and Roberts 2020). Isotope samples were collected from 154 different locations (Fig. 1). Some locations were sampled only once whereas others were sampled up to 30 times to evaluate temporal variation in isotope compositions. The sites sampled most were the American Tunnel, Gold King mine, Mogul mine, Natalie Occidental mine and Red & Bonita mine. Of the 154 sites sampled for isotopes, 95 sites were sampled at least four times and sample collection was generally dispersed throughout the year to include both high- and low-flow periods. Samples of ${\text{δ}}^{2}\text{H}$ and ${\text{δ}}^{18}\text{O}$ analysis were collected unfiltered in cleaned 25-ml borosilicate bottles with no-headspace to avoid evaporation or fractionation. Laboratory analysis was completed using L1102-i isotopic liquid wavelength-scanned cavity ring down spectroscopy. Results of isotopic analysis are reported in per mil (‰) relative to Vienna Standard Mean Ocean Water (V-SMOW). Analytical uncertainties were 1.0‰ for ${\text{δ}}^{2}\text{H}$ and 0.5‰ for ${\text{δ}}^{18}\text{O}$.

Data from previous watershed scale evaluations conducted by Church et al. (2007) and collected between 1997 and 2000 were compiled from Sole et al. (2007). These datasets included metals and REEs analysed by inductively coupled plasma atomic emission spectrometry (ICP-AES) as described by Mast et al. (2007). This dataset contains samples collected from 167 locations including mines, streams and springs, and locations are illustrated in Figure 1.

Following the designation of the Bonita Peak Mining District Superfund Site, samples for REEs were collected by the MSI in 2019 and 2020. These samples were collected from 181 distinct locations representing springs, mines and streams, some of which were also sampled during the studies described in Church et al. (2007; Fig. 1). Analysis of metals and REE samples collected by the MSI was conducted at the EPA Office of Research and Development laboratory in Ada, Oklahoma. Rare earth elements were measured in field-filtered samples (0.45 μm) using high-resolution inductively coupled plasma mass spectrometry (HR-ICP-MS). Samples were preserved with high-purity HNO_3_ (2%). Field quality control samples included field blanks and field duplicates. The HR-ICP-MS method and associated quality control procedures were described in Wilkin et al. (2021). Results of quality control testing for field samples and laboratory control samples from this study are presented and discussed in the Supplementary material (Table S1). Preliminary analysis of REE signatures and geospatial variation within the study area was conducted by Dorsk (2020).

Compositions of REEs were normalized to the North American Shale Composite (NASC) according to values summarized in Gromet et al. (1984). Normalization to the NASC permits recognition of patterns within REE compositions (Noack et al. 2014). In addition to pattern evaluation, quantitative REE metrics were calculated for europium anomaly (Eu/Eu*), cerium anomaly (Ce/Ce*) and enrichment in middle REE $\left(E_{\text{MREE}}\right)$. Both Eu/Eu* and Ce/Ce* were calculated according to equations in Noack et al. (2014) and $E_{\text{MREE}}$ was calculated according to equations in Pérez-López et al. (2010):

(1) $$\text{Ce}/{\text{Ce}}^{*}=\frac{2{\text{Ce}}_{\text{NASC}}}{{\text{La}}_{\text{NASC}}+{\mathrm{Pr}}_{\text{NASC}}};$$

(2) $$\text{Eu}/{\text{Eu}}^{*}=\frac{2{\text{Eu}}_{\text{NASC}}}{{\text{Sm}}_{\text{NASC}}+{\text{Gd}}_{\text{NASC}}};$$

(3) $$E_{\text{MREE}}=\frac{Y_{\max}}{Y_{\text{o}}}-1$$

Here, subscripts NASC indicate NASC-normalized concentrations and $Y_{\max}$ and $Y_{\text{o}}$, respectively, indicate the maximum polynomial curve and straight line derived from curve fitting of NASC-normalized concentrations of Nd, Sm, Eu, Dd, Tb and Dy. See Pérez-López et al. (2010) for additional information on calculation of $E_{\text{MREE}}$.

Both Eu/Eu* and Ce/Ce* are useful potential metrics because they may record interactions between water and specific lithologies or alteration types (Göb et al. 2013) and are potentially indicative of redox processes (Noack et al. 2014). The quantity $E_{\text{MREE}}$ was shown to be useful for quantifying progressive sulfide oxidation and identifying AMD-affected waters (Pérez-López et al. 2010). Rare earth elements have been used in the study area previously in an effort to quantify pre-mining water quality (Mast et al. 2007) and in a comparison study with other mining districts (Verplanck et al. 1999). These previous studies indicated that most waters in the study area are enriched in middle REEs (MREEs; Sm, Eu, Gd, Tb and Dy) when compared with light REEs (LREEs; La, Ce, Pr and Nd) and heavy REEs (HREEs; Ho, Er, Tm, Yb and Lu), and that some springs displayed negative Ce/Ce*, which was likely linked to redox processes (Verplanck et al. 1999; Mast et al. 2007). These studies did not directly assess the influence of alteration style on REE signatures tracing metal transport processes. Potential influence of alteration assemblages was evaluated in this study by assigning alteration to sampling locations using surficial alteration mapping from satellite-based remote sensing (Dalton et al. 2007) combined with detailed geological mapping (Bove et al. 2007). The alteration assemblage immediately underlying each sample point was assigned to the sample location. All stable isotope and REE data compiled for this analysis are published and available in Newman et al. (2024).

The quantitative metrics Ce/Ce*, Eu/Eu* and $E_{\text{MREE}}$ were tested for statistically significant differences between alteration styles using *t*-tests. Although the raw REE data are highly non-parametric, the NASC normalization and anomaly calculation process yielded Ce/Ce*, Eu/Eu* and $E_{\text{MREE}}$ values that were normally distributed based on results of a Kolmogorov–Smirnov (KS) test, a hypothesis test used to evaluate normality (Helsel et al. 2020). Because Ce/Ce*, Eu/Eu* and $E_{\text{MREE}}$ values are normally distributed, *t*-tests are appropriate for evaluating differences (Helsel et al. 2020). The null hypothesis of each *t*-test is that the groups being compared are not significantly different from one another. The alternative hypothesis is that the groups being compared are significantly different from one another. When the *P*-value was less than 0.05, the null hypothesis was rejected.

## Results and discussion

### Stable isotopes and groundwater flow paths

**Fig. 2.**
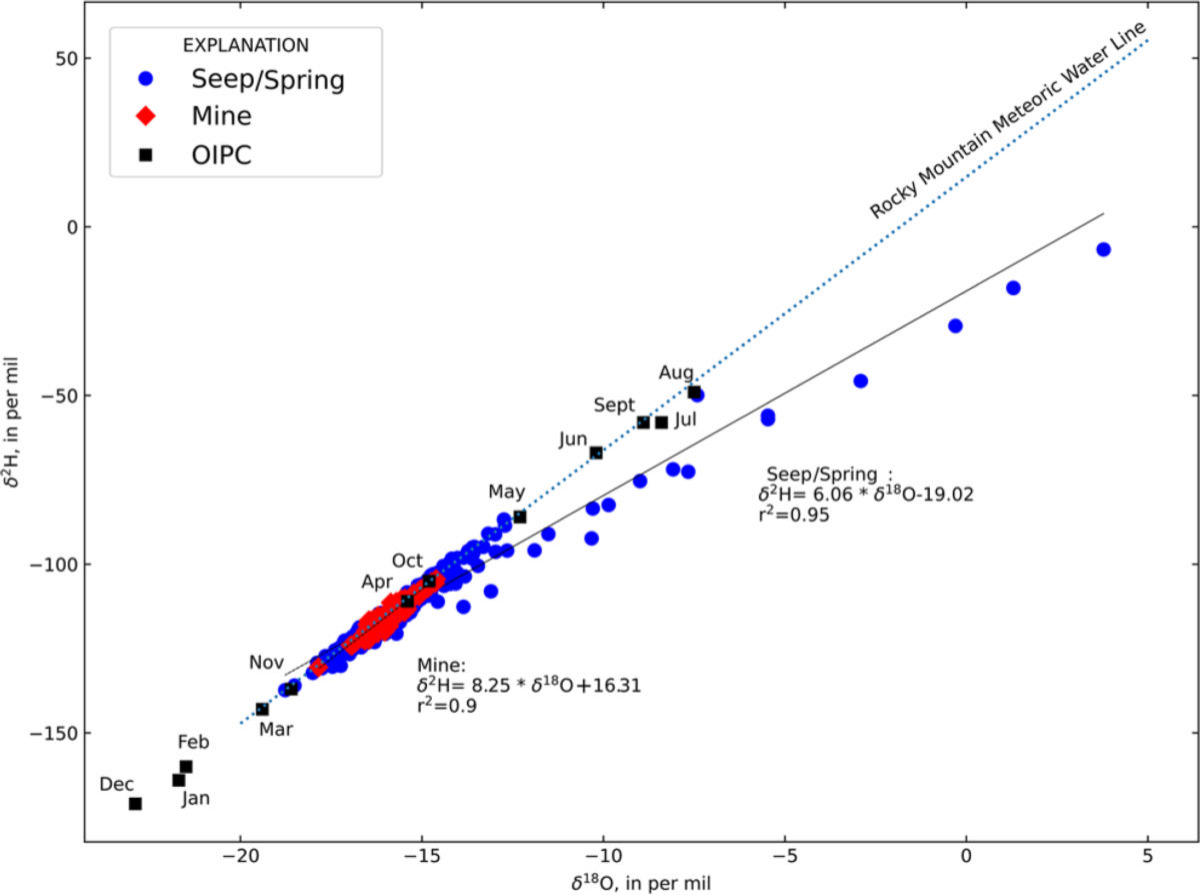
Stable isotopic compositions of mines and springs. Source: the RM-MWL is derived from Anderson et al. (2016). Estimates of the isotopic composition of precipitation are derived from the Online Isotopes in Precipitation calculator for Bonita Peak (Bowen 2024) and are labelled by month.

Stable isotopic compositions of mines and springs are shown in Figure 2 relative to the Rocky Mountain meteoric water line (RM-MWL) derived from Anderson et al. (2016) and estimates of isotopic composition of precipitation from each month of the year derived from the Online Isotopes in Precipitation calculator (Bowen 2024) for the summit of Bonita Peak (37.888 °N, −107.621 °W, 4045 m elevation). Utilizing estimates of isotopic composition of precipitation may produce some uncertainty given the potential high variability of precipitation in mountain regions (Jasechko 2019), but there are no proximal locations with observations of isotopes in precipitation.

Springs have stable isotopic compositions ranging from −18.77 to 3.78 ‰ and −137.31 to −6.73 ‰, respectively, for ${\text{δ}}^{18}\text{O}$ and ${\text{δ}}^{2}\text{H}$. Mines display a narrow isotopic range from −17.85 to −14.61 ‰ and −130.54 to 104.44 ‰, respectively, for ${\text{δ}}^{18}\text{O}$ and ${\text{δ}}^{2}\text{H}$. Best-fitting lines indicate that the composition of the mines is consistent with the RM-MWL (Table S2) and is most similar to estimates of precipitation compositions from October/November and March/April. However, direct identification of snowmelt recharge components by month using estimates of precipitation isotopes alone neglects possible fractionation during snowmelt processes (Earman et al. 2006). The best-fitting line for springs indicates some deviation from the RM-MWL and has a shallower slope indicative of minor evaporation. Minor evaporation in some springs could be caused by spring morphology where spring pools with large surface area to volume ratios or relatively small groundwater inflow rates allow for evaporative enrichment in ${\text{δ}}^{2}\text{H}$ and ${\text{δ}}^{18}\text{O}$.

**Fig. 3.**
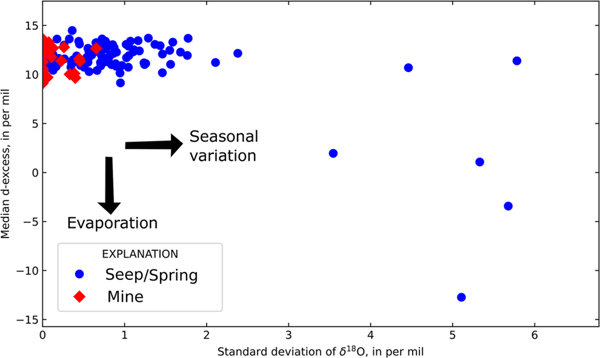
Plot of standard deviation of ${\text{δ}}^{18}\text{O}$ v. median d-excess from locations with four or more samples through time.

Stable isotopic compositions of mines show less variability than springs in ${\text{δ}}^{2}\text{H}$ and ${\text{δ}}^{18}\text{O}$ space (Fig. 2) and have a distinctive pattern when considering the standard deviation of ${\text{δ}}^{18}\text{O}$ v. median deuterium excess (d-excess; Fig. 3), a quantity useful for evaluating evaporation. Negative d-excess values indicate greater evaporative isotopic fractionation (Jasechko 2019). Mines have low standard deviations of ${\text{δ}}^{18}\text{O}$ and have no evidence of evaporation. In contrast, springs display a wide range in ${\text{δ}}^{18}\text{O}$ standard deviation and some spring locations display substantial evaporative fractionation. Greater ${\text{δ}}^{18}\text{O}$ standard deviation in springs indicates that some of these features may be recharged by shorter duration events such as summer rainstorms. There are two likely causes for the low variability in isotopic composition of mines: (1) mines are preferentially recharged during a shorter period (making their isotopic compositions more homogeneous), or (2) mines are representative of a larger volume of groundwater storage that is well mixed. It is likely that the latter is responsible for the relatively invariant composition of mines when compared with springs because both mines and springs are present within the same watersheds, and therefore it is likely that both are recharged by similar processes. Also, because some mine workings are developed deep into the subsurface, it is possible that mines with more extensive or deeper workings are intercepting water from multiple distinct zones of groundwater storage within the mountain system (Cowie and Roberts 2020).

**Fig. 4.**
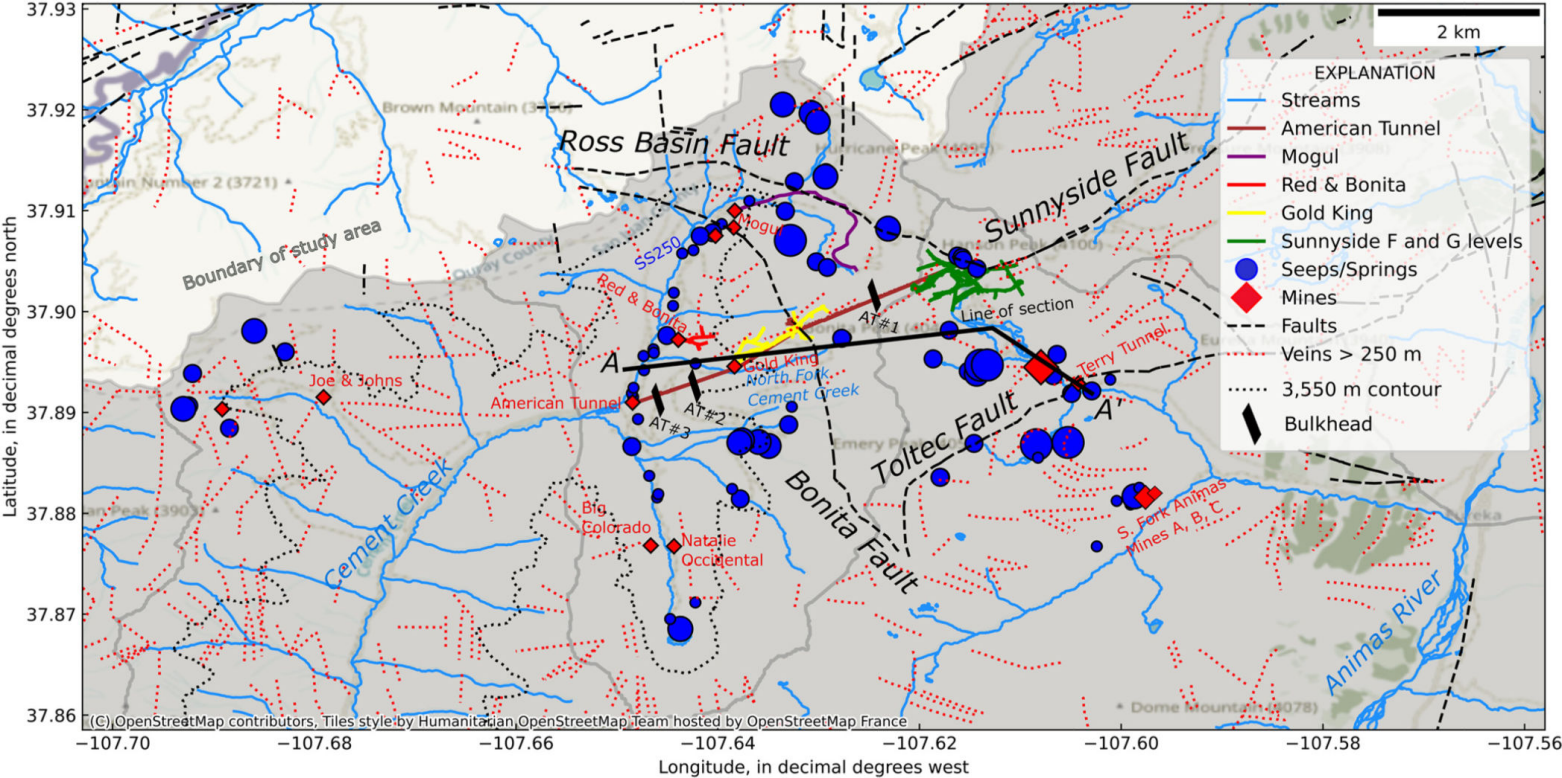
Map of standard deviation of ${\text{δ}}^{18}\text{O}$ in mines and springs. Symbol size is scaled to standard deviation with larger symbols indicating sites with larger standard deviation. Only veins with strike length greater than 250 m are shown. Line of section (A–A’) for Figure 9 is shown. See Figure 1 for location within larger study area. Source: faults and veins are from Sole et al. (2007).

Spatial variation in the standard deviation of ${\text{δ}}^{18}\text{O}$ (Fig. 4) illustrates that seeps and springs in the Upper Cement Creek watershed, which contains the American Tunnel, Gold King, Mogul and other draining mines, tend to have a lower standard deviation of ${\text{δ}}^{18}\text{O}$ than adjacent watersheds. This is particularly evident for springs adjacent to Cement Creek. Springs in the former (now drained because of sudden lake collapse into the mine; Jones 2007) Lake Emma Basin (above the Sunnyside mine workings and between the Sunnyside and Toltec Faults) and Ross Basin (east of the Mogul mine along the Ross Basin Fault) generally show the highest standard deviations whereas springs in Prospect Gulch (to the NW of Cement Creek near the Joe & Johns mine) display moderate standard deviations.

**Fig. 5.**
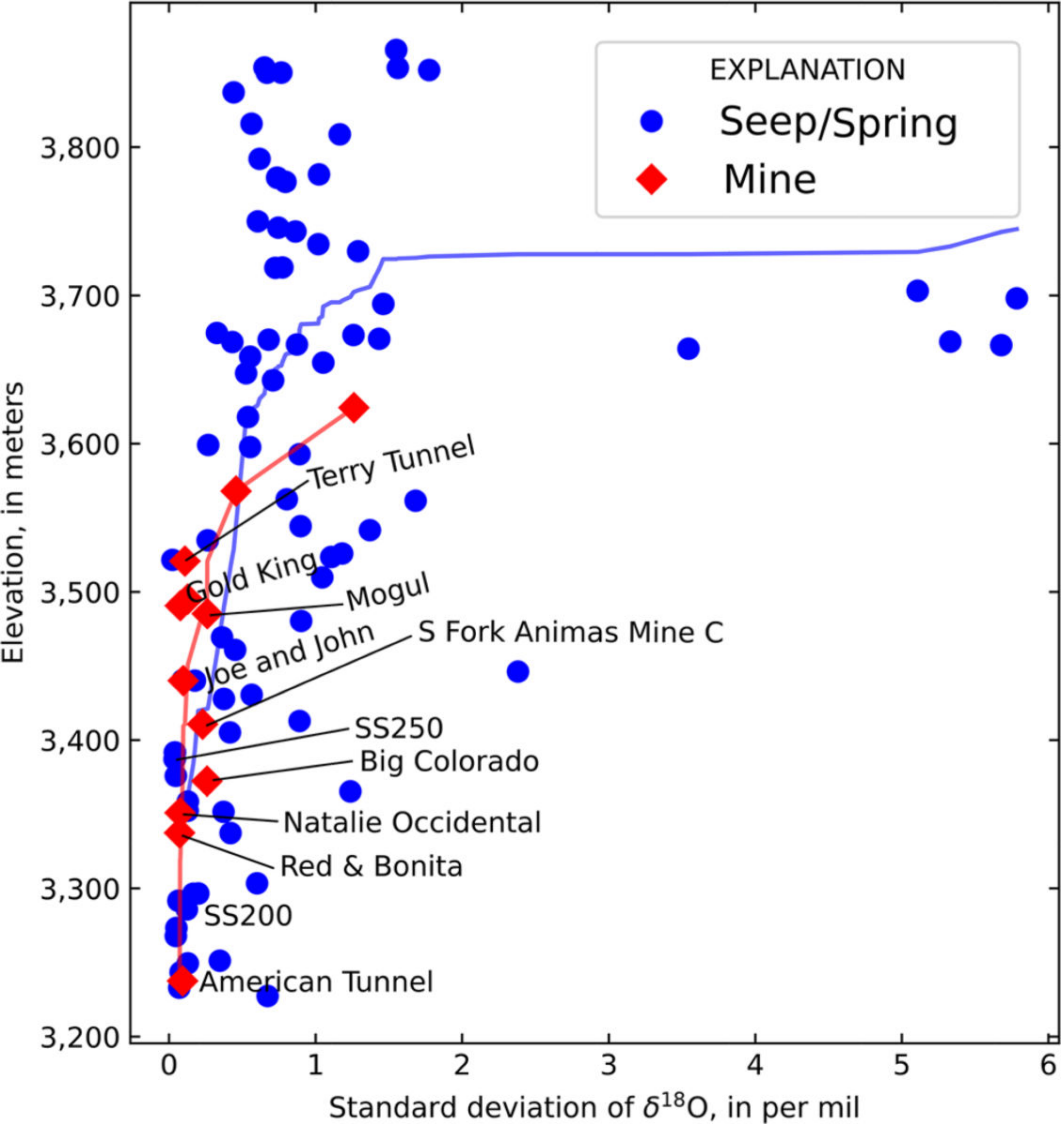
Plot of standard deviation of ${\text{δ}}^{18}\text{O}$ v. elevation (in North American Vertical Datum of 1988) for mines and springs with four or more records. Sites of interest are labelled and each dataset is shown with a loess line, an approach that accounts for potential outliers by approximating the central behaviour of the dataset (Helsel et al. 2020).

Given the apparent spatial variation in the standard deviation of ${\text{δ}}^{18}\text{O}$ with elevation, relationships between site elevations and the standard deviation of ${\text{δ}}^{18}\text{O}$ were evaluated (Fig. 5). Both springs and mines tend to have greater standard deviations at higher elevations, and loess lines, smoothed locally fit lines that represent the central tendency of the datasets with respect to potential outliers (Helsel et al. 2020), have similar form below approximately 3550 m elevation (Fig. 5). Many of these springs and mines with similar behaviour below 3550 m elevation are located with the Upper Cement Creek watershed (Fig. 4). This elevation is similar to the pressure head measured at the AT #1 bulkhead of 3557 m in 2002 (Sorenson and Brown 2015). Notably, several mines that are distant from each other spatially, yet at similar elevations, display similar standard deviations of ${\text{δ}}^{18}\text{O}$. Examples include the Gold King and Mogul mines (approximately 1.7 km apart) and the Red & Bonita and Natalie Occidental mines (approximately 2.3 km apart). Similarity across elevation bands also extends beyond watershed divides. The Terry Tunnel displays a similar standard deviation of ${\text{δ}}^{18}\text{O}$ to the Gold King and Mogul, yet is located across the watershed divide in the Lake Emma basin (Fig. 4). Even more distant, the South Fork of the Animas Mine C plots close to the loess line yet is not spatially associated with any of the other mines (Fig. 4), the closest mine being the Terry Tunnel at 1.4 km distance (but in a different subwatershed). There are no known mine workings connecting the vicinity of South Fork of the Animas Mine C to any mines near or connected to the Sunnyside mine. South Fork of the Animas Mine C is unique in this respect because other mines in the Lake Emma basin and Upper Cement Creek are connected by tunnels or located within the same watershed (Fig. 4). The similarity in stable isotopic signatures below 3550 m elevation and the correspondence of this value with pressure head measurements from the AT #1 bulkhead indicate that many of the springs and mines below this elevation may be deriving groundwater from a singular, well-mixed source. The median ${\text{δ}}^{18}\text{O}$ v. elevation (Fig. S1) was also evaluated given the common assumption that stable isotopes may be used to trace recharge elevation (Jasechko 2019). In the study watershed, there was no relationship between median ${\text{δ}}^{18}\text{O}$ and elevation, likely because mines and large springs are capturing recharge from a wide range of elevations that results in a well-mixed isotopic signal.

The American Tunnel shows one of the smallest standard deviations of ${\text{δ}}^{18}\text{O}$ of any mine and is located at the lowest elevation. The American Tunnel also displays similar ${\text{δ}}^{18}\text{O}$ values to the Gold King and Red & Bonita mines (Fig. S2) despite being 100–250 m lower in elevation than those mines, with more depleted isotopic compositions in these locations compared with the Mogul and Natalie Occidental mines. During mining, the American Tunnel served as the primary drainage for the Sunnyside and Gold King mines, keeping these mines largely dry during various phases of operation (Jones 2007). The modern isotopic signal of drainage from the American Tunnel, with minor seasonal variation and depleted compositions, supports the hypothesis that this feature still drains much of the overlying bedrock, thereby integrating seasonal and interannual isotopic variations and producing a low-amplitude pattern with low standard deviation. Bulkheads within the American Tunnel likely contribute to how this feature acts to control the regional groundwater flow, given that the last observed pressure head at bulkhead AT #1 was 3557 m (Sorenson and Brown 2015). Both springs and mines below this elevation display lower seasonal variation in ${\text{δ}}^{18}\text{O}$ (Figs 4 and 5), indicating that water impounded behind this bulkhead may be discharging to the adjacent fractured bedrock groundwater system.

### Rare earth elements and metal sources

**Fig. 6.**
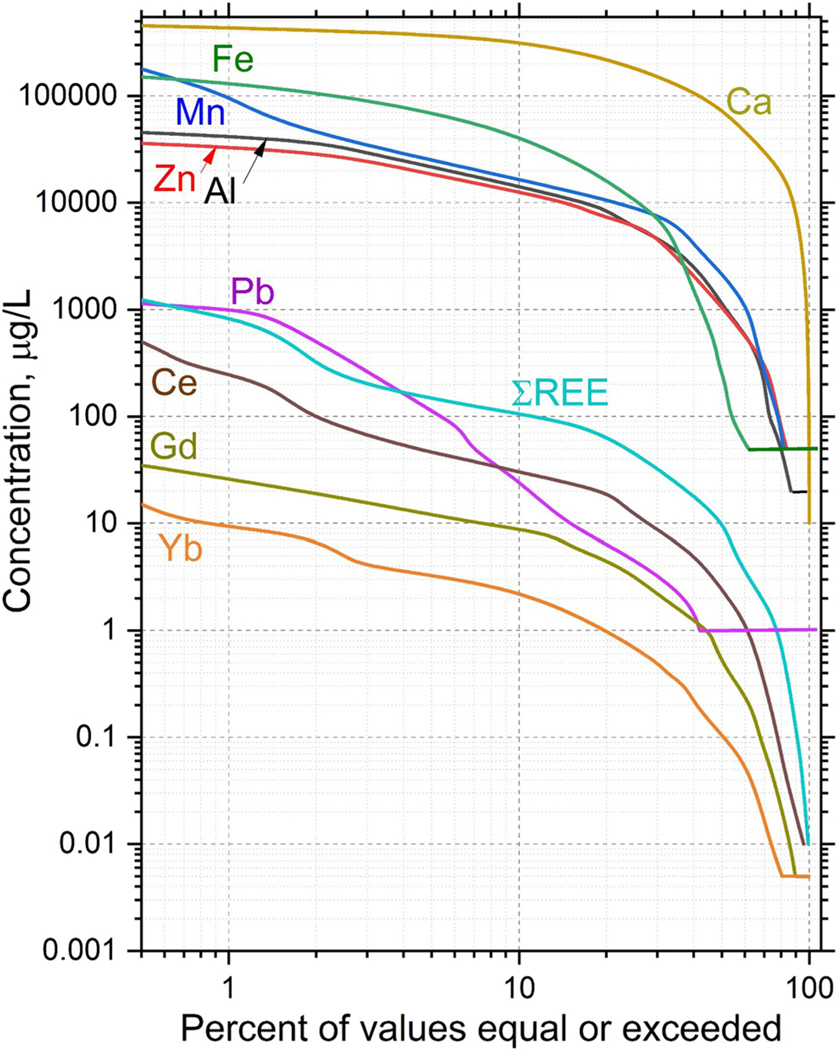
Cumulative distributions of dissolved REEs compared with selected major and trace metals from samples collected in 2019–2020. Samples from all types of locations (springs, mines and streams) are shown. Horizontal line segments represent the quantitation level for the individual elements.

Cumulative distribution functions of REE concentrations compared with other major and trace metals indicate that Ca, Fe and Mn are the most abundant metals in the aqueous phase, consistent with observations from many other AMD-affected sites (Fig. 6; Göb et al. 2013; Duraz et al. 2023). Aluminium and Zn are the next most enriched metals in the watershed, and Zn represents one of the primary constituents of ecological concern (Walton-Day et al. 2021). The sums of all REEs have a similar abundance to Pb at high concentrations, but at moderate to lower concentrations; the total REEs and Ce alone become more abundant than Pb. Although REEs have lesser concentrations than other metals, they show similar overall patterns in Figure 6, with the exceptions of Mn and Pb which have steeper slopes in the middle region of the plot. The largely similar patterns between total REEs, Ce, Gd and Yb when compared with Fe, Zn and Al allow the REEs to be used as general indicators of metal sourcing in the study area. The REEs can be used as a group to identify geochemical processes such as sorption, sulfide oxidation and secondary mineral precipitation (Göb et al. 2013; Grawunder et al. 2018; Liu et al. 2024) and these processes may be obscured or difficult to quantify using concentrations of Fe, Zn and Al alone. Because of the utility of REEs in elucidating these varied processes in mineralized watersheds and the similarity of REE distributions with constituents of interest such as Al, Fe and Zn (Fig. 6), REEs can be used to better understand metal sources in the Upper Animas River watershed.

**Fig. 7.**
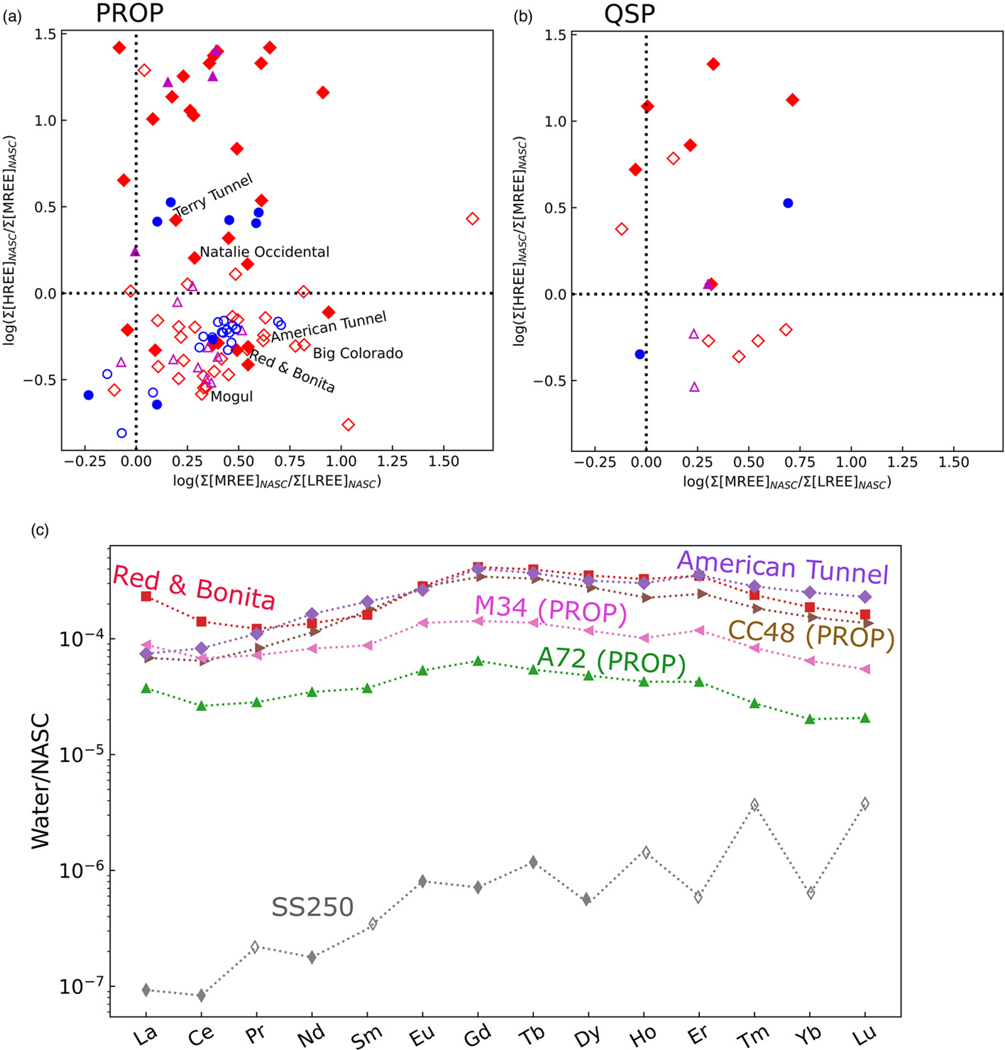
Normalization diagrams for alteration types: (**a**) PROP; (**b**) QSP; (**c**) spider diagram for selected sites. On panels (a) and (b) sites with pH <5 are shown by open symbols and sites with pH >5 are shown by filled symbols. All sample locations shown in panel (c) are less than 3500 m in elevation. In panel (c), open symbols are used to represent censored results less than the detection limit, which only occurred for the sample from SS250.

The range of NASC-normalized REE compositions in the study area are shown on a normalization diagram in Figure 7, where Figures 7a and b illustrate the ratio of NASC-normalized MREEs over LREEs on the *x*-axis and NASC-normalized HREEs over MREEs on the *y*-axis. As described by Noack et al. (2014), normalization plots provide an effective means for visualizing various REE patterns in a simple biplot.

The majority of sampling locations are located within PROP alteration (Fig. 7), which is the most abundant alteration style in the study area (Bove et al. 2007; Mast et al. 2007; Yager et al. 2013). From a mineralogical perspective, sites located in PROP alteration should have substantial acid neutralizing capacity given abundant calcite and subsidiary chlorite and epidote, which have lower acid neutralizing capacity than carbonates (Sherlock et al. 1995). In general, the trend of PROP alteration and acid neutralization was noted by previous studies, wherein Yager et al. (2013) found that watersheds dominated by PROP alteration typically showed circumneutral pH at their outlets, compared with watersheds dominated by QSP or AS, which tended to have acidic pH at their outlets. However, surficial alteration style does not fully account for alteration assemblages that groundwater flow paths may encounter within the subsurface.

The potential complexity of flow paths and subsurface interaction with a variety of alteration types is illustrated in Figure 7a because many of the sampling locations located within PROP alteration are characterized by acidic pH (as shown by open symbols). These PROP samples with acidic pH, from springs, draining mines and streams, are nearly all found in the bottom-right quadrant of the normalization diagram, indicating enrichment in MREEs consistent with previous investigations of AMD in Colorado (Verplanck et al. 1999; Mast et al. 2007) and in Europe (Pérez-López et al. 2010). Most of the primary mines of environmental concern (American Tunnel, Red & Bonita, Mogul) are located within surficial PROP alteration. The locations with PROP alteration and acidic pH are generally located in the Upper Cement Creek watershed (Fig. S3). In contrast to acidic samples, locations within surficial PROP alteration with circumneutral pH nearly all plot in the upper-right quadrant, where HREEs and MREEs have similar proportions. Such samples are generally located outside the Upper Cement Creek watershed (Fig. S3). Geochemical patterns observed in REEs could also be explained by geological differences, but the majority of sampling locations are located within the Silverton Volcanics (Fig. S4; Yager and Bove 2007) and therefore do not represent differing bedrock geology. In this instance, the REEs are a useful indicator of potential geochemical complexities arising from interactions in the subsurface because of the differentiable groupings of samples from PROP locations, which have likely been affected by metal mobilization from rock with alteration assemblages other than PROP. In this manner, the REE geochemistry of these locations records subsurface weathering that is not accounted for by mapping surficial alteration alone.

Samples found in other alteration styles are less abundant than PROP but tend to follow the pattern observed in PROP wherein acidic samples are more enriched in MREE compared with circumneutral samples. Figure 7c illustrates a NASC-normalized spider diagram of REE compositions from several of the locations of interest illustrated in Figures 7a and b. Notably, the American Tunnel and Red & Bonita mine display similar REE patterns even though one is acidic and the other circumneutral (Fig. 7a). The primary difference is that the circumneutral Red & Bonita has greater NASC-normalized concentrations of LREEs La and Ce and lesser NASC-normalized concentrations of HREEs Tm, Yb and Lu. Samples from SS250 (location shown in Fig. 4) illustrate the dilute composition of this spring, which is located within surficial QSP alteration but has a circumneutral pH (Fig. 7b), and is not enriched in MREEs compared with HREEs. The sample from SS250 also illustrates artefacts of the detection limits for REEs in this dilute sample, as evident by the alternating spiked pattern. This pattern is caused by the Oddo–Harkins effect, wherein elements with even atomic numbers are more abundant in the universe than elements with odd atomic numbers (Noack et al. 2014). In this sample, Ce and Nd are above the detection limit whereas Pr and Sm are less than the detection limit (which was used for plotting purposes). The constituents most affected by concentrations less than detection limits were Lu and Tm with, respectively, 56% and 34% of samples less than detection limits. The detection limits and potential artefacts with NASC normalization do not affect the results of this study because the quantities using NASC-normalized REE concentrations (Ce/Ce*, Eu/Eu* and $E_{\text{MREE}}$; equations 1, 2 and 3) are calculated using constituents that were above detection limits in 87% to 95% of the samples (e.g. Ce, Eu, Gd, La). The majority of samples in this study were acidic, and REE solubility is increased at lower pH (Fig. S6; Noack et al. 2014). Therefore, although concentrations below detection limits in some dilute samples cause visual patterns in the NASC-normalized REE concentrations (Fig. 7c), the quantitative and statistical analyses of these datasets are unaffected.

Samples from Cement Creek above the confluence with the Animas River (CC48), from Mineral Creek above the confluence with the Animas River (M34) and from the Animas River below Silverton (A72) all illustrate the MREE-enriched signature common to most waters in the study area that have been influenced by AMD (Fig. 7c; locations of all three sites shown on Fig. 1). Mineral Creek (location M34) displays a similar REE signature to Cement Creek (CC48), despite none of the large, flooded mines considered in this study being present in Mineral Creek.

The similarity of A72 with draining mines and CC48 (locations shown on Fig. 1) suggests that the MREE signature could be used as a tracer to understand the downstream migration of the AMD signature (Olías et al. 2018). The extent of downstream migration of the AMD signature derived from draining mines in the Upper Animas River watershed could be utilized to understand the net environmental effect of historical mining in the headwaters of the San Juan River compared with other sources of metals in the watershed. As tributaries enter the river downstream of the Silverton caldera, the REE signature could be modified by dilution, sorption and co-precipitation (Pérez-López et al. 2010; Olías et al. 2018). Potential additional research could evaluate the downstream extent of the REE signature specific to metal sourcing within the Upper Animas River watershed by collecting synoptic samples along the Animas and San Juan Rivers.

Given the potential uniqueness of REE signatures in different alteration styles (Pérez-López et al. 2010; Göb et al. 2013) and in waters of varying acidity (Fig. 7; Noack et al. 2014), the quantitative REE metrics Ce/Ce*, Eu/Eu* and $E_{\text{MREE}}$ were each examined using hypothesis tests for the potential to provide additional information pertinent to metal sourcing and transport. Because two distinctive populations of samples collected from PROP alteration are evident (Fig. 7a), this alteration style was further categorized based on observed pH: we use PROP–Acid to indicate samples collected from surficial PROP alteration but with pH less than 5 whereas PROP–Neut indicates samples collected from surficial PROP alteration that displayed pH greater than 5.

Results of statistical comparisons are summarized in Table 2. The results indicate that each metric displays some statistically significant differences between alteration styles. It appears that Ce/Ce* may be the most useful for geochemical discrimination as this metric has six pairs of alteration styles with significant differences. Samples of PROP locations display significant differences in both Ce/Ce* and $E_{\text{MREE}}$ depending on their pH (compare PROP–Acid v. AS to PROP–Neut v. AS and PROP–Neut v. QSP in Table 2), indicating that both Ce/Ce* and $E_{\text{MREE}}$ may be useful metrics by which to evaluate the sourcing of metals in AMD environments (Pérez-López et al. 2010). Both of these metrics may be complicated in watersheds where both AMD and natural sulfide oxidation co-occur (Nordstrom 2015), and the analyses that utilize REE metrics could include stable or radiogenic isotopes (Mathur et al. 2013; Kidder et al. 2021), geochemical modelling (Kimball et al. 2010) and other mass balance approaches (Mast et al. 2007) to differentiate between AMD and naturally derived metals. In addition to the statistically significant differences between PROP–Acid and PROP–Neut, it is worth noting that PROP–Acid does not display significant differences with QSP, one of the primary alteration styles associated with AMD in the watershed (Bove et al. 2007). The lack of statistical difference between PROP–Acid and QSP could indicate that groundwater is interacting with subsurface QSP (or other acid-generating alteration styles) before discharging at the surface within the PROP assemblage. The comparison between PROP–Acid and QSP is pertinent because QSP is one of the most abundant alteration styles within the Sunnyside mine workings (Casadevall and Ohmoto 1977), which form much of the mine complex that potentially generates discharge to mine adits and springs within the Cement Creek watershed. Based purely on acid-base accounting, all locations within PROP alteration would be expected to produce circumneutral pH (Yager et al. 2008) and REE signatures that differed from acid-generating alteration assemblages such as QSP. These signatures therefore provide an example that using surficial mineralogy and alteration assemblages alone may be an oversimplification for classification of water-quality signatures and metal sources. In addition to existing geological and alteration complexity, the excavation of mine workings may also complicate the approach of using REE signatures alone to trace alteration styles.

### Defining groundwater flow and transport processes

The primary goal of this analysis is to utilize ${\text{δ}}^{2}\text{H}$, ${\text{δ}}^{18}\text{O}$ and REEs to better characterize groundwater flow paths and processes that govern metal mobilization and transport in the Upper Animas River study area. These constituents are ideal for this purpose because ${\text{δ}}^{2}\text{H}$ and ${\text{δ}}^{18}\text{O}$ are direct tracers of the water molecule, thereby providing information on the physical processes of groundwater recharge, storage, flow and discharge (Jasechko 2019; Wolkersdorfer et al. 2020; Langman et al. 2023), whereas Ce and Eu effectively record water–rock interactions and redox processes along a flow path (Noack et al. 2014; Grawunder et al. 2018; Liu et al. 2024), though these approaches have limitations (e.g. Cendón et al. 2022). This analysis leverages data collected by multiple investigations over more than a 20-year period and spanning multiple smaller watersheds and alteration styles.

Stable isotopic variation indicates multiple nested orders of groundwater flow paths in the study area (i.e. Tóth 1963). High elevation springs have high seasonal variability in stable isotopic signature, evident from standard deviations of ${\text{δ}}^{18}\text{O}$ (Fig. 5) and experience some evaporation prior to recharge or in spring pools as indicated by d-excess values (Fig. 3). Many of these high elevation springs are located within young alluvial deposits (QU), which likely have low storage volume and transmit water relatively rapidly. High elevation springs also generally have circumneutral pH and highly variable REE signatures, indicating relatively minor sulfide oxidation and subsequent metal loading along short groundwater flow paths. These high elevation springs are likely representative of short flow paths that originate and discharge in a single watershed.

Springs and mines at lower elevations display lower standard deviations of ${\text{δ}}^{18}\text{O}$ and d-excess values indicating no evaporative fractionation during recharge (Figs 3–5). The lack of temporal variation in isotopic compositions in these locations is consistent with longer transit times and greater mixing within a large subsurface storage volume. The relationship of standard deviation of ${\text{δ}}^{18}\text{O}$ and increased mixing was investigated using hypothetical incremental mixing:

(4) $$\delta^{18}O_{\text{mix}}=\left(F_{n}*\delta^{18}O_{n}\right)+\left(F_{n+1}*\delta^{18}O_{n+1}\right)+\cdots +\left(F_{i}*\delta^{18}O_{i}\right)$$

(5) $$1=F_{n}+F_{n+1}+\cdots +F_{i}$$

**Fig. 8.**
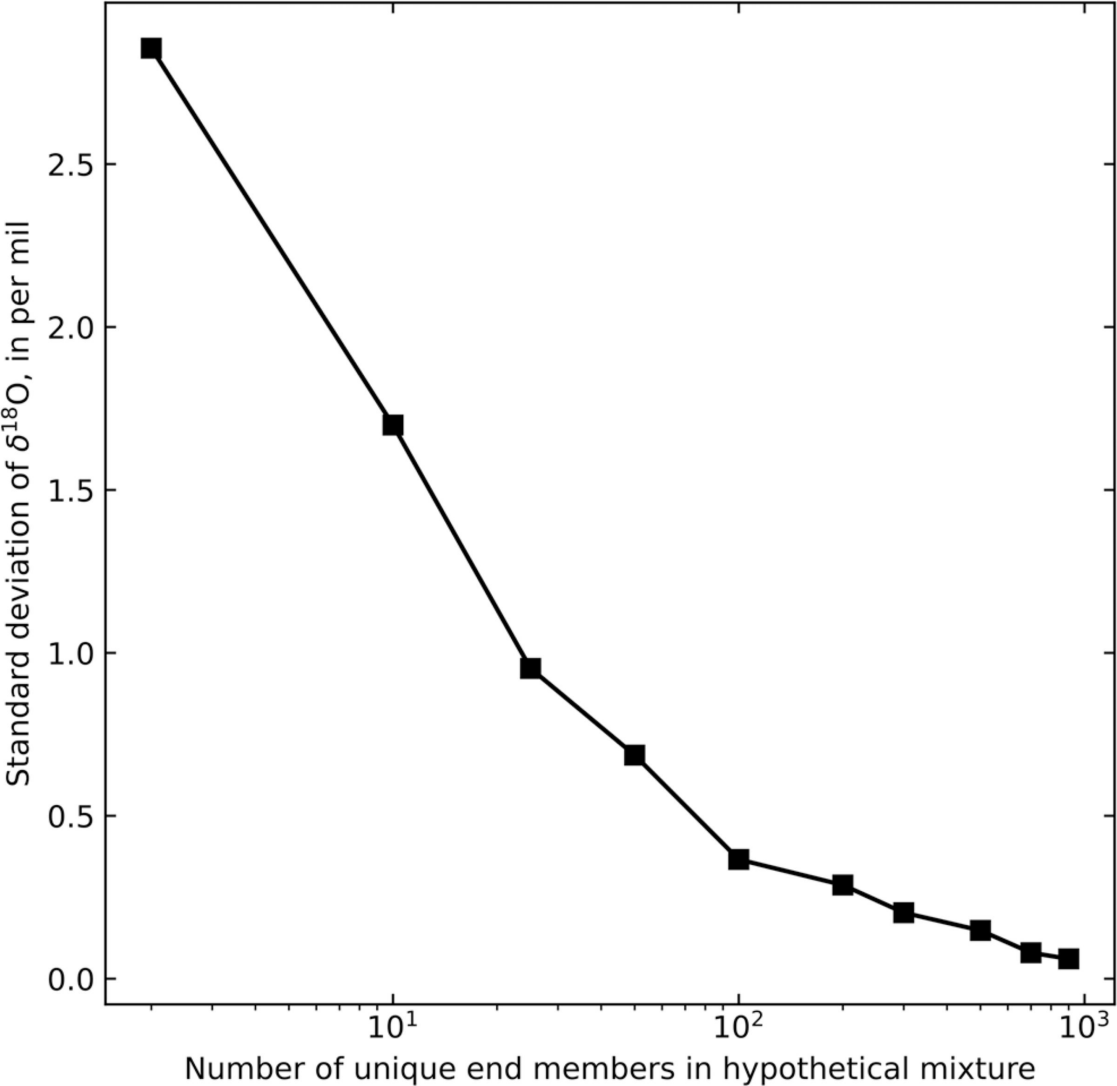
Results of hypothetical mixing calculations between end members derived from modern precipitation estimated using OIPC (Bowen 2024).

Here, ${\text{δ}}^{18}{\text{O}}_{\text{mix}}$ is the mixed oxygen isotopic composition, $F_{n}$ is the fraction of the nth end member in the mixture, ${\text{δ}}^{18}{\text{O}}_{n}$ is oxygen isotopic composition of the nth end member in the mixture, $F_{i}$ is the fraction of the final ith end member in the mixture and ${\text{δ}}^{18}{\text{O}}_{i}$ is oxygen isotopic composition of the final ith end member in the mixture. Numbers of end members (values of i) were considered between 2 and 900, and each value of ${\text{δ}}^{18}{\text{O}}_{n}$ was selected from a random normal distribution created from the estimates of local precipitation from OIPC (Bowen 2024). For each i value, the process was repeated 20 times, and the standard deviation of those 20 iterations was used to calculate the hypothetical standard deviation of ${\text{δ}}^{18}\text{O}$ resulting from mixing of the given number of end members. Results of this hypothetical incremental mixing are illustrated in Figure 8, wherein the hypothetical standard deviation of ${\text{δ}}^{18}\text{O}$ shows an inverse correlation with the number of end members. This illustrates that the standard deviation of ${\text{δ}}^{18}\text{O}$ metric is useful for conceptually understanding groundwater mixing, because as the aquifer is increasingly well mixed, the standard deviation of ${\text{δ}}^{18}\text{O}$ of discharge from the aquifer (springs and mines in this study) becomes increasingly homogenized. Locations with few numbers of end members may be exemplified by springs with seasonal recharge during spring snowmelt and fall rainstorms, whereas locations with numerous end members may include deep mine tunnels that experience near continuous groundwater recharge from precipitation of widely varying composition.

**Fig. 9.**
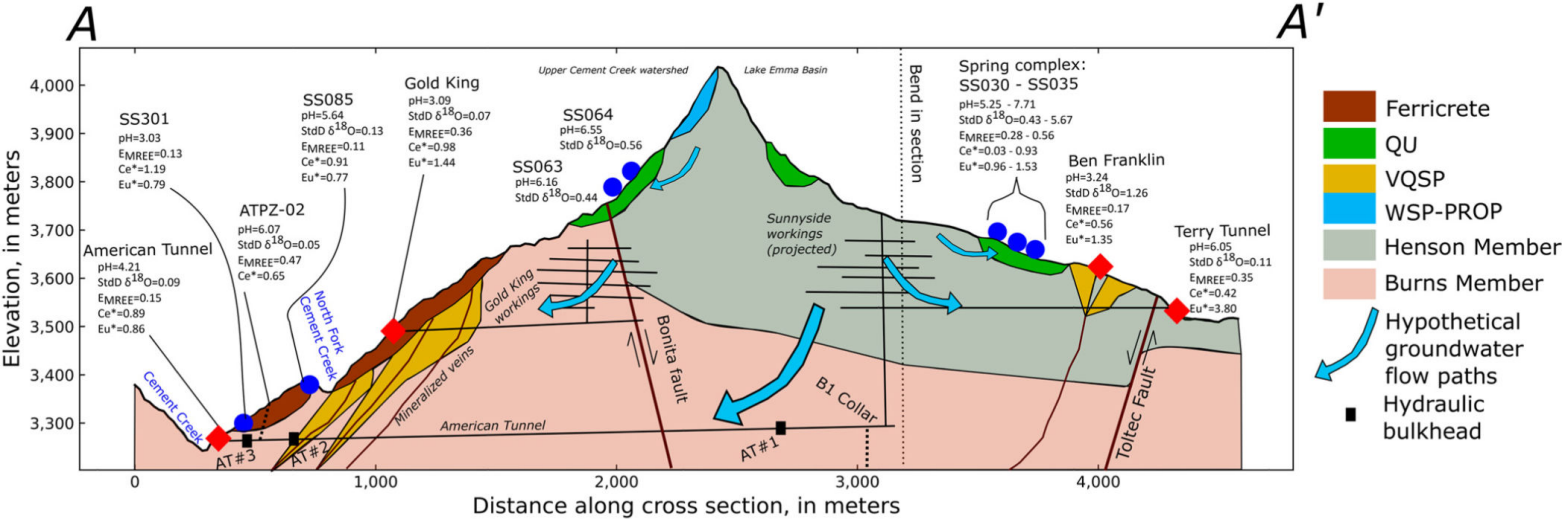
Hydrogeological conceptual cross-section along line A–A’ (Fig. 4) displaying bulkhead locations (Walton-Day et al. 2021), surficial alteration styles (Bove et al. 2007), ferricrete distribution (Yager et al. 2007), geological units (Burbank and Luedke 1969), faults and mineralized veins (Burbank 1951; Burbank and Luedke 1969; Casadevall and Ohmoto 1977) and underground mine workings (Burbank 1951; Casadevall and Ohmoto 1977; Guzman et al. 2023). Surface topography derived from USGS digital elevation model on 20 m node spacing. Thickness of ferricrete and locations of Sunnyside mine workings (upper levels) are schematic only. Geochemical characteristics of sampled springs (blue circles) and mines (red diamonds) near the cross-section are displayed; where single values are displayed (i.e. pH, Ce/Ce*, etc.) this represents a single sample from fall 2018 or fall 2019. Ranges are used to represent multiple sampling locations in close proximity. Vertical exaggeration = 2.15, elevation relative to North American Vertical Datum of 1988.

The highest density of springs and mines displaying a low standard deviation of ${\text{δ}}^{18}\text{O}$ (consistent with greater mixing) is along Cement Creek between the Bonita Fault and the American Tunnel and along South Fork Cement Creek (Fig. 4). Being at lower elevation and close to first- and second-order streams (Fig. 9), these springs and mines may be receiving a mixture of locally derived flow paths and flow paths that originate in smaller, higher-elevation watersheds (Tóth 1963; Gleeson and Manning 2008). It is also possible that interbasin groundwater flow occurs from the Lake Emma Basin (east side of watershed divide) to Upper Cement Creek watershed (west side of divide; Fig. 9), which was one of the remediation goals of bulkhead installation (Walton-Day et al. 2021). Previous investigations hypothesized that before mining, the interbasin flow from Lake Emma Basin to Cement Creek was promoted by the 10°–14° dip to the SW of the Burns and Henson Members of the Silverton Volcanics (Simon Hydro-Search 1993).

Evidence for potential interbasin groundwater flow is also provided by the prevalence of ferricrete, an iron oxide cemented clastic deposit, in the Upper Cement Creek compared with Lake Emma Basin (Yager et al. 2007). Ferricrete is common along much of Cement Creek, South Fork Cement Creek and North Fork Cement Creek. It requires specific conditions to form, namely an interface between upwelling iron-rich acidic groundwater and flowing oxic surface water. These conditions promote precipitation of iron oxyhydroxides, which form the cement for ferricrete (Wirt et al. 2007). Maps of surficial alteration illustrate that the Cement Creek watershed is dominated by PROP alteration, which is not generally acid-generating. Some occurrences of acid-generating alteration such as AS are present on the western boundary of the watershed, with minor occurrences of VQSP on the eastern slopes (Bove et al. 2007). The frequency of acid springs (Fig. S3) and ferricrete (Yager et al. 2007) in the Upper Cement Creek watershed is therefore somewhat surprising. What is the source of metals and acidity for these springs, when surficial alteration mapping would indicate that the watershed should create circumneutral pH drainage (Yager et al. 2013)?

Alteration and sulfide mineralization in the Silverton caldera is spatially complex and varies both vertically and horizontally as erosion has revealed multiple levels of the hydrothermal plumbing system (Casadevall and Ohmoto 1977; Bove et al. 2007; Anderson et al. 2023). Although most of the Upper Cement Creek watershed is dominated by PROP at the surface, it is possible that VQSP (as shown along North Fork Cement Creek; Bove et al. 2007) and other acid-generating alteration styles are more prevalent in the subsurface. An alternative hypothesis is that the excavation of mine workings within Upper Cement Creek watershed has increased subsurface oxidation of sulfides disseminated through the PROP alteration, resulting in generation of acidity that would not have occurred without anthropogenic disturbance. Estimation of the age of ferricrete provides evidence that upwelling of iron-rich and acidic groundwater has been occurring in Upper Cement Creek watershed for up to 8000 years (Verplanck et al. 2007). Although the age of ferricrete does not preclude additional subsurface acid generation due to anthropogenic activities, it indicates that upwelling acidic groundwater has discharged to Upper Cement Creek watershed long before mining. The source of this acidic upwelling groundwater could be either locally derived or the result of interbasin groundwater flow. Given the circumneutral pH of headwater springs in the watershed (Fig. 9) and the general lack of surficial acid-generating material (Bove et al. 2007; Yager et al. 2013), it is considered likely that deeply circulating interbasin groundwater flow is interacting with sulfide mineralization or acid-generating altered rock at depth before these flow paths discharge to surface water.

Additional evidence of potential metal sourcing in the deep subsurface is provided by REE signatures. Similarity of $E_{\text{MREE}}$ in lower elevation mine tunnels and springs suggests a common source for dissolved REEs. The Terry Tunnel and ATPZ-02 (an inclined monitoring well drilled into the American Tunnel) display elevated $E_{\text{MREE}}$ (ranging from 0.35 to 0.47; Fig. 9; Table S3). These high $E_{\text{MREE}}$ values are consistent with sulfide-bearing rock from the Sunnyside mine described in Casadevall and Ohmoto (1977) and sampled from a borehole that was drilled downward from within the Sunnyside workings (borehole B1), which has diagnostically high $E_{\text{MREE}}$ (Fig. S5). The similarity of $E_{\text{MREE}}$ in these two sample sites (located in different watersheds; Fig. 9), and the fact that both $E_{\text{MREE}}$ values are anomalously high even in this AMD-affected watershed, could be explained by both locations deriving REEs from the flooded Sunnyside mine workings (which are located primarily below the Lake Emma Basin; Fig. 9). The difference in $E_{\text{MREE}}$ between the American Tunnel drainage $\left(E_{\text{MREE}}=0.15\right)$ and ATPZ-02 is likely attributable to different oxidation conditions between the sampling location within the tunnel and ATPZ-02. Flow sampled from the American Tunnel is near the surface and not immediately adjacent to the outer bulkhead (AT #3). Contrastingly, ATPZ-02 is completed between bulkheads AT #2 and AT #3 and is representative of reducing conditions within the flooded mine workings. Channelized flow within the mine tunnel under near atmospheric conditions may allow ${\text{Eu}}^{2+}$ to oxidize to ${\text{Eu}}^{3+}$, and be adsorbed or coprecipitate (Noack et al. 2014), before being sampled at the mouth of the American Tunnel, modifying the $E_{\text{MREE}}$ signature representative of the deeper flooded mine workings. Oxidation is also indicated by the differing pH of ATPZ-02 v. the American Tunnel, where oxidation of dissolved ferrous Fe and hydrolysis between these two sampling locations likely results in acidic pH sampled from the mine adit. This study reaffirms the utility of $E_{\text{MREE}}$ in tracing groundwater flow paths and water–rock interactions that has been indicated by previous studies on AMD.

Although this investigation provides evidence that interbasin groundwater flow and deep subsurface sourcing of metals may be occurring in the study area, additional investigations could be conducted to provide further evaluation of these processes. As described by Gleeson and Manning (2008) and Haitjema and Mitchell-Bruker (2005), the likelihood of interbasin groundwater flow may be assessed using a combination of hydraulic conductivity, recharge rates and watershed dimensions. Data exist to estimate hydraulic conductivities for the area (Simon Hydro-Search 1993; Johnson 2007) and recharge could be estimated using vertical temperature profiles in new monitoring wells completed in 2023 (Anderson 2005) or using geochemical approaches (Crosbie et al. 2018). Environmental tracers such as noble gases (Manning and Caine 2007), groundwater residence times (McCallum et al. 2020) and stable isotopes of sulfate (Kim et al. 2020) and metals (Mathur et al. 2013) could also be used to place further constraints on groundwater circulation and metal transport. Additional evaluation of structural controls on groundwater flow, such as the prevalence of faults and veins as potential conduits or barriers to flow, could also improve understanding of the study area. Early geological mapping of the area (Burbank 1951; Burbank and Luedke 1969) displays greater density of mineralized veins and structures in the vicinity of North Fork Cement Creek than are shown by Bove et al. (2007). Focused structural evaluation of the area, paired with geochemical sampling, could improve remedial decision-making given the potential importance of the Gold King mine in contributing solutes downstream (Rodriguez-Freire et al. 2016) and uncertainty in subsurface connections between the Gold King, Red & Bonita, American Tunnel, and other mines in the watershed.

This study also defines the REE character of AMD derived from the Upper Animas River. Because of geochemical processes such as sorption, coprecipitation and simple dilution, it is unlikely that the REE signature of AMD from the Upper Animas is transported along the full length of the river until it discharges to the San Juan River. Although conservative transport is unlikely, longitudinal sampling specifically designed to evaluate the spatial extent of downstream AMD transport could be utilized to evaluate how metals derived from the Upper Animas River are transported and attenuated.

## Conclusions

The Upper Animas River watershed is heavily affected by AMD and forms one of the headwaters for the San Juan River. Understanding metal sourcing and mobility in the Upper Animas River watershed may therefore have a bearing on quantifying effects on downstream water users along the San Juan River. Conceptual evaluation of groundwater flow and metal transport is also pertinent to remedial decisions made within the Bonita Peak Mining District Superfund Site. This study utilized stable isotopes of water combined with REEs to evaluate sources of groundwater recharge and discharge and metal mobilization within this complex mountain watershed.

Stable isotopes sampled from springs display seasonal variation that is likely linked to temporal changes in recharge source. In contrast, most mines display seasonally stable compositions of stable isotopes, as indicated by the standard deviation of ${\text{δ}}^{18}\text{O}$. This indicates that most mines are discharging water from a large volume of groundwater storage that is well mixed. Consistency in stable isotope signatures from mines in adjacent watersheds also indicates that they could be connected to the same or similar subsurface storage. This study illustrates the utility of temporal measurements of stable water isotopes in defining groundwater mixing, and shows that groundwater that is more well mixed could be expected to display a relatively consistent character (as quantified by the standard deviation of ${\text{δ}}^{18}\text{O}$).

Signatures of REEs from various alteration styles show statistically significant variations and illustrate that surficial alteration mapping alone may not account for sulfide oxidation in the subsurface that imparts some springs and draining mines with substantial enrichment in MREEs. This MREE enrichment is consistent with previous studies of the watershed (Verplanck et al. 1999; Mast et al. 2007) and other areas with AMD globally (Pérez-López et al. 2010; Grawunder et al. 2014). Enriched MREE signatures throughout the study area, including in the Animas River as it exits the watershed, indicate that this signature could be used to trace the extent of the downstream migration of AMD, and the influence of processes that affect metal mobility such as sorption, coprecipitation and dilution. This approach has been shown to be effective in other watersheds with AMD (Olías et al. 2018) and could be utilized in further studies within the San Juan River.

Although this study provided various insights into the hydrologic and geochemical functioning of the Upper Animas River watershed, some additional questions remain. Additional studies could leverage the existing monitoring network and decades of data to further explore questions of subsurface connectivity of mine workings and the effects of mining on metal transport. Such potential studies could lend insight into remedial decision-making in the study area and provide information important to the characterization of other complex sites.

## Supplementary Material

Supplementary Material

## Figures and Tables

**Table 1.** Summary of datasets utilized in this analysis

Data type	Date range	Methodology	References
Stable isotopes of water	2016–2020	Isotopic liquid wavelength scanned cavity ring down spectroscopy	Cowie and Roberts (2020)
Trace metals and rare earth elements	1997–2000	Inductively coupled plasma atomic emission spectrometry	Mast et al. (2007); Sole et al. (2007)
Trace metals and rare earth elements	2018–2020	High-resolution inductively coupled plasma mass spectrometry	Dorsk (2020)

All datasets are compiled into Newman et al. (2024).

**Table 2.** Results of paired t-tests between alteration styles

	PROP–Acid	PROP–Neut	QU	AS	QSP	VQSP	WSP–PROP
Eu/Eu*							
PROP–Acid	1.000						
PROP–Neut	0.059	1.000					
QU	**0.009**	0.605	1.000				
AS	0.947	0.501	0.269	1.000			
QSP	0.451	0.297	0.158	0.709	1.000		
VQSP	0.185	0.570	0.544	0.428	0.554	1.000	
WSP–PROP	0.745	0.679	0.457	0.629	0.617	0.510	1.000
Ce/Ce*							
PROP–Acid	1.000						
PROP–Neut	**<0.001**	1.000					
QU	0.166	0.264	1.000				
AS	**0.021**	**0.002**	**0.046**	1.000			
QSP	0.655	**0.013**	0.339	**0.019**	1.000		
VQSP	0.955	0.081	0.446	0.221	0.751	1.000	
WSP–PROP	0.616	0.173	0.398	0.528	0.415	0.756	1.000
$E_{\text{MREE}}$							
PROP–Acid	1.000						
PROP–Neut	**<0.001**	1.000					
QU	0.055	0.428	1.000				
AS	0.246	0.282	0.739	1.000			
QSP	0.902	**0.007**	0.172	0.363	1.000		
VQSP	**0.036**	0.859	0.754	0.608	0.158	1.000	
WSP–PROP	0.427	0.146	0.340	0.339	0.562	0.442	1.000

Statistically significant (*P*-value <0.05) results are highlighted in bold text. PROP–Acid = propylitic alteration with acidic pH; PROP–Neut = propylitic alteration with circumneutral pH; QU = quaternary undifferentiated; AS = acid sulfate; QSP = quartz–sericite–pyrite; VQSP = vein-related quartz–sericite–pyrite; WSP–PROP = weak sericite–pyrite and propylitic.

## Data Availability

The datasets generated during and/or analysed during the current study are available in the USGS ScienceBase repository, https://doi.org/10.5066/P9OOHY1Q.
