# Supplementary material for: Tracing metal sources and groundwater flow paths in the Upper Animas River watershed using rare earth elements and stable isotopes
Source: Geochemistry (Bath). Author manuscript; Available in PMC 2026 May 5. (PMC13137996; doi:10.1144/geochem2024-023)
Supplement: Supplementary Material [file NIHMS2056348-supplement-Supplementary_Material.docx]

**Supplementary File for: Tracing metal sources and groundwater flow paths in the Upper Animas River watershed using rare earth elements and stable isotopes**

Connor P. Newman ^1, 2^ (ORCID: [0000-0002-6978-3440](https://orcid.org/0000-0002-6978-3440))*, Rory Cowie ^3^_,_ Richard T. Wilkin ^4^, Alexis Navarre-Sitchler ^2^

^1^ U.S. Geological Survey, Colorado Water Science Center, Denver, CO., USA

^2^ Colorado School of Mines, Geochemistry Program, Department of Geology and Geological Engineering, Golden, CO., USA

^3^ Alpine Water Resources LLC, Silverton, CO., USA

^4^ U.S. Environmental Protection Agency, Office of Research and Development, Groundwater Characterization and Remediation Division, OK

*Corresponding author: [cpnewman@usgs.gov](mailto:cpnewman@usgs.gov)

Any use of trade, firm, or product names is for descriptive purposes only and does not imply endorsement by the U.S. Government.

# Laboratory methods and quality assurance

Rare earth element (REE) concentrations were measured using High Resolution-Inductively Coupled Plasma-Mass Spectrometry (HR-ICP-MS; Thermo Element XR) in medium- and high-resolution modes to resolve potential isobaric interferences (Wilkin et al., 2021). Custom-made interference check standards containing mixtures of Pr, Nd, Eu, Sm, Gd, Tb, and Ba were analyzed with sample sequences to evaluate mass resolution and confirm the accurate correction of oxide interferences (e.g., ^135^Ba^16^O on ^151^Eu). Rhodium (^103^Rh; 1 µg/L, Inorganic Ventures) was used as an internal standard to correct for instrumental drift. Minimum detection limits for the REEs ranged from about 1 to 4 ng/L. Both filtered (0.45-micron) and unfiltered samples were collected. This study focuses on an analysis of the filtered REE samples. Samples were preserved with nitric acid in the field at the time of sample collection. Duplicate field samples to assess reproducibility were randomly collected at about 10% of the sample locations. Samples were shipped on ice and stored in a laboratory refrigerator prior to analysis. Quality control (QC) samples in the laboratory included lab replicates, lab blanks, matrix spikes, quantitation limit checks, calibration check standards, interference checks, and second-source standards.

Five-point calibrations including a calibration blank were prepared in 2% high-purity HNO_3_ at the following concentrations: 0.02 μg/L, 0.1 μg/L, 0.5 μg/L, and 2 μg/L for all REE. Each run of 20 unknown samples consisted of one second-source standard (2 μg/L of La, Ce, Pr, Nd, Sm, Eu, Gd, Tb, Dy, Ho, Er, Tm, Yb, and Lu, High Purity Standards) and lab blank pair analyzed immediately after calibration, four continuing calibration verification (2 μg/L of La, Ce, Pr, Nd, Sm, Eu, Gd, Tb, Dy, Ho, Er, Tm, Yb, and Lu, Inorganic Ventures) and lab blank pairs distributed throughout the analytical sequence, 1 low level quantitation limit check standard, 2 sample replicates, 1 serial dilution, 2 matrix spikes, and a series of 3 interference check solutions. Linear dynamic range (LDR) was checked with a 100 μg/L standard (90-110% recovery); any sample with a concentration above the LDR was diluted accordingly. The quantitation limit (QL) was set at 0.02 μg/L (for undiluted samples). Minimum detection limits were based on the standard deviation of 10 replicate runs of a 5 ng/L solution.

QC measures obtained in multiple (19) runs for dissolved REEs are described in **Table S1**. Data quality objectives were: 90-110% recovery for the second-source standard, 90-110% recovery for continuous calibration verification checks, <QL for the method blanks, 70-130% recovery for low level QL checks (at 0.02 μg/L), relative percent difference <20% and <30% for lab and field replicates, respectively, and 75-125% recovery for matrix spikes. The QC evaluations were intended to evaluate instrument stability, data accuracy and precision, potential for bias by interferences and matrix issues, and sources of potential bias from sample contamination. A total of 3921 element-specific quality control tests were conducted and 97.3% of the tests met predetermined data quality control objectives. In cases where data quality objectives were not met, the analytical data were evaluated, and it was determined if sample reanalysis was required. The data quality control results (**Table S1**) indicated that the REE data were suitable to meet project objectives.

Other metals plotted in Figure 6 of the primary manuscript are Al, Zn, Mn, Fe, Ca, and Pb. All of these metals except Pb were analyzed using Inductively Coupled Plasma – Optical Emission Spectrometry (Perkin Elmer Optima DV ICP-OES) following EPA Method 200.7 (U.S. Environmental Protection Agency, 1994a). Quality control samples included second source standards, continuing calibration verification samples, method blanks, interference check samples, serial dilutions, lab replicates, low-level QL checks, matrix spikes, and matrix spike replicates. Lead concentrations were measured using HR-ICP-MS in medium resolution mode following general practices described in EPA Method 200.8 (U.S. Environmental Protection Agency, 1994b); QL = 1 μg/L). Quality control samples included second source standards, continuing calibration verification samples, method blanks, interference check samples, serial dilutions, lab replicates, low-level QL checks, and matrix spikes. All metals concentrations reported here were from field-filtered (0.45-micron) and acid-preserved samples. All data are available in Newman et al. (2024).

**Table S1.** QA/QC tests for REE analyses, test purpose, and summary of results for each QC sample type.

| **QA/QC Test & Purpose** | **Results** |
| --- | --- |
| Second Source Standard: a test solution prepared from a source different from the source of the calibration standards; run immediately after calibration (2 μg/L, all REEs). | 19 second source standard tests were analyzed (266 elements) with an average recovery of 99.7% (min 84.8%; max 110%). Six tests were outside of the expected range (90-110% recovery) by <5%. |
| Continuing Calibration Verification (CCV): the purpose of the CCV tests is to ensure that the calibration of the instrument is valid and stable through an analytical sequence. 4 CCVs were run with each set of 20 samples; CCVs were prepared at 2 μg/L for each REE. | 68 CCV analyses were analyzed with an average recovery of 102.9% (range 86.2 to 117.3%). 51 of 952 individual element tests were outside of control (90-110% recovery) by less than 7%. No expected data quality impact. |
| Initial Calibration Blank & Continuing Calibration Blanks: blank samples that do not contain the analytes of interest at a detectable level. Run after the second source standard and CCV checks. | 87 method blanks were analyzed. All method blanks were below the QL, except one blank with La and Ce above the QL, probably due to carry over from a high concentration sample. No expected data quality impact. |
| Low Level QL Checks (LLQL): test solution prepared at or near the QL. Each sequence of 20 samples contained one LLQL at 0.02 μg/L for each REE. | 19 LLQL samples were analyzed with an average recovery of 105.5% recovery (min 70%; max 125%). All LLQL checks were within control limits (70-130%). |
| Laboratory Replicates: replicates serve as a test of instrument stability, and consistency in laboratory sample preparation and analysis. | 35 Lab Replicates were tested. Comparisons were made using relative percent differences (RPDs) when both the primary and duplicate sample had an REE concentration >5x the QL. A total of 266 comparisons were made with an average Relative Percent Difference of 2.9% (min 0.1%; max 24.3%). Two of 266 lab Duplicate comparisons were outside of control limits (RPD<20%) by <4%. |
| Matrix Spikes: Matrix spike tests are conducted by adding a known amount (a spike) of analyte to a sample, testing the spiked sample, and determining if the analyte is recovered; used to evaluate sample matrix issues. | 26 matrix spikes (420 elements tested) were analyzed with an average Percent Recovery of 93.5%. 34 of 420 MS tests were outside of control, indicating possible matrix issues or non-ideal spike levels. |
| Interference Check Samples: Isobaric interferences were tested using custom element standards. | 48 interference check samples were analyzed. Eu was outside of control by <0.05 μg/L in 3 tests and Pr and Nd were outside of control in 2 tests. No expected data quality impact. |
| Field Blanks assess potential contamination introduced from sample containers with applicable preservative and ambient sources of contamination. | 15 field blanks were collected and analyzed(210 element tests). 85% of the field blanks showed no-detectable REEs. Of the 210 checks, 32 and 2 detections were above the MDL (0.002 μg/L) and QL (0.02 μg/L), respectively. |
| Sample Replicates represent precision of field sampling, analysis, and site heterogeneity. | 28 field replicates were collected and analyzed. Comparisons were made (RPDs) when both the primary and duplicate sample had an REE concentration >0.01 μg/L. The mean RPD was 6.4% (min 0%; max 126%). 1.8% of the field replicates had an RPD>30%. |

# Stable isotopes

**
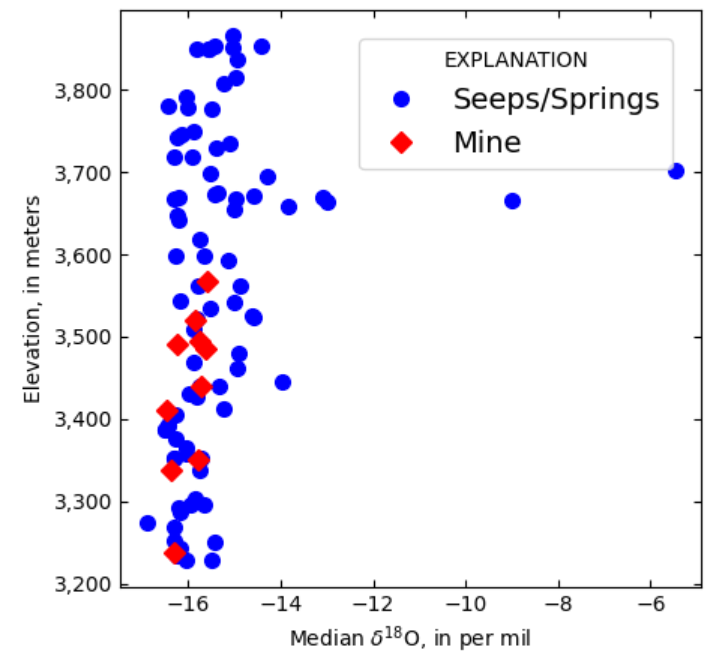
**

**Fig. S1.** Elevation versus median δ^18^O.


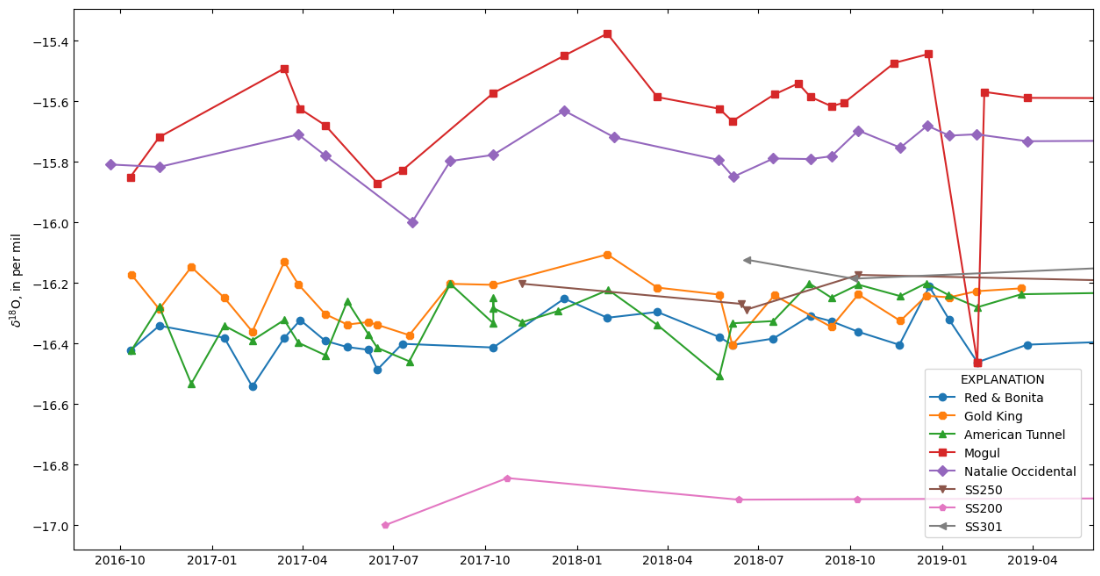


**Fig. S2.** Timeseries of δ^18^O in selected springs and draining mines in the Upper Cement Creek watershed.

**Table S2.** Results from Online Isotopes in Precipitation Calculator (OIPC; Bowen, 2024) for the summit of Bonita Peak (37.888 °N, -107.621 °W, 4,045 m elevation). V-SMOW = Vienna Standard Mean Ocean Water.

| **Month** | **δ^2^H (‰, V-SMOW)** | **δ^18^O (‰, V-SMOW)** |
| --- | --- | --- |
| Jan | -101 | -13.3 |
| Feb | -90 | -12.1 |
| Mar | -85 | -11.3 |
| Apr | -53 | -7.4 |
| May | -30 | -4.5 |
| Jun | -16 | -2.6 |
| Jul | -18 | -2.6 |
| Aug | -12 | -2.2 |
| Sept | -15 | -2.7 |
| Oct | -51 | -7.4 |
| Nov | -66 | -9.3 |
| Dec | -107 | -14.4 |

# Spatial and geochemical associations in rare earth elements


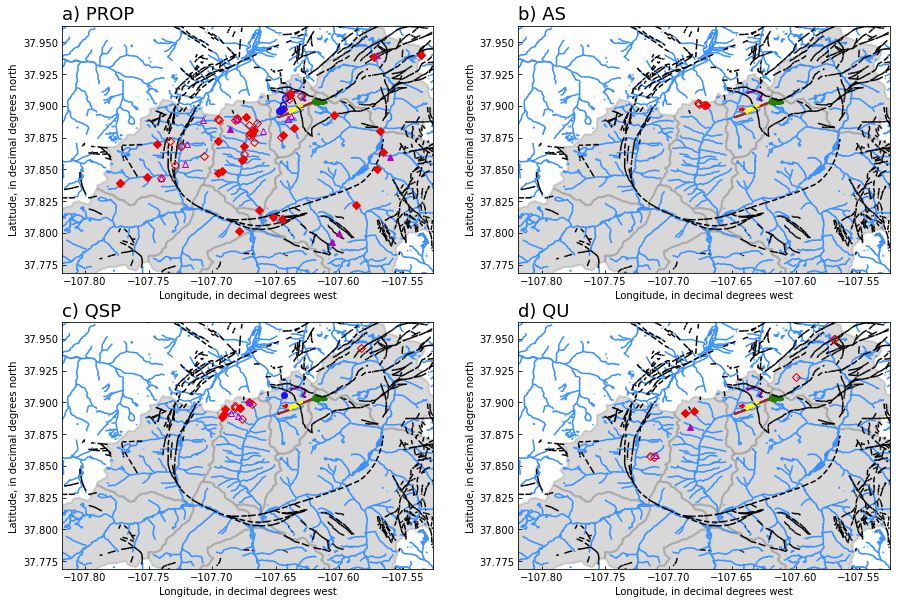


**Fig. S3.** Map of site locations in different alteration styles indicated by pH, open symbols indicate locations with pH less than 5 and filled symbols indicate locations with pH greater than 5. See primary manuscript figure 1 for symbology. Draining mines are located within the Upper Cement Creek watershed.


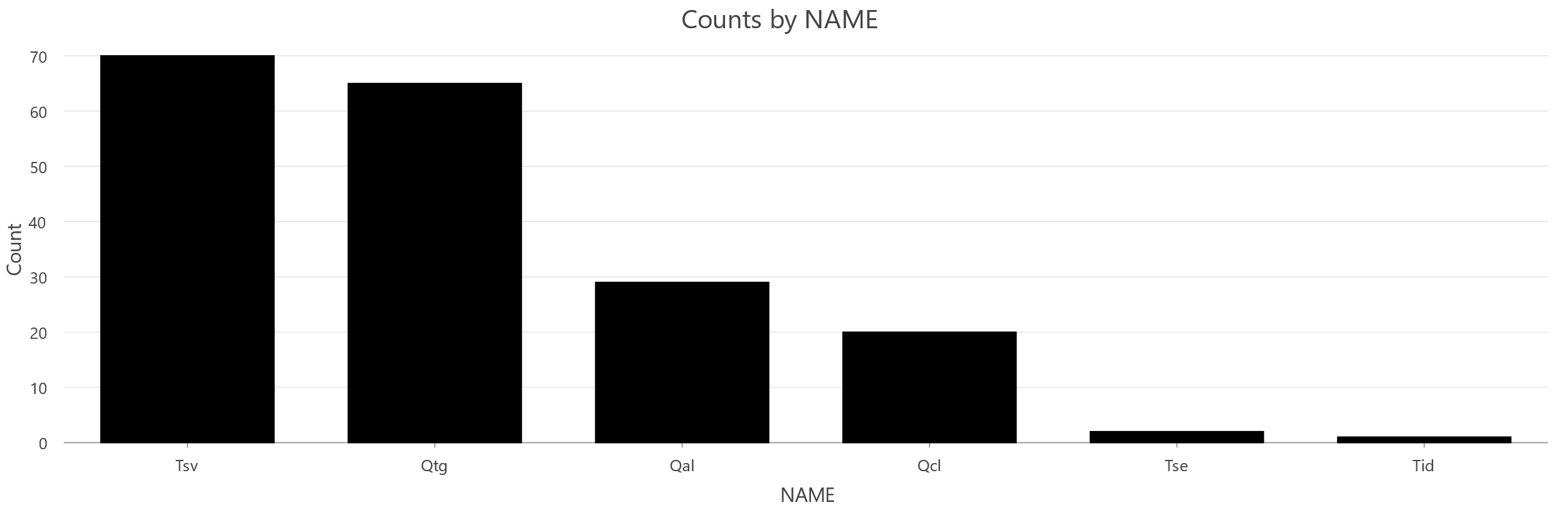


**Fig. S4.** Histogram of geologic classifications of sampling locations determined using sample coordinates and geology described in Yager and Bove (2007). Tsv = Silverton Volcanics; Qtg = Talus, glacial deposits, and rock glaciers; Qal = Alluvium; Qcl = Colluvium and landslide deposits; Tse = Eureka Member and Picayune Megabreccia Member of Sapinero Mesa Tuff; Tid = Dacite intrusions.


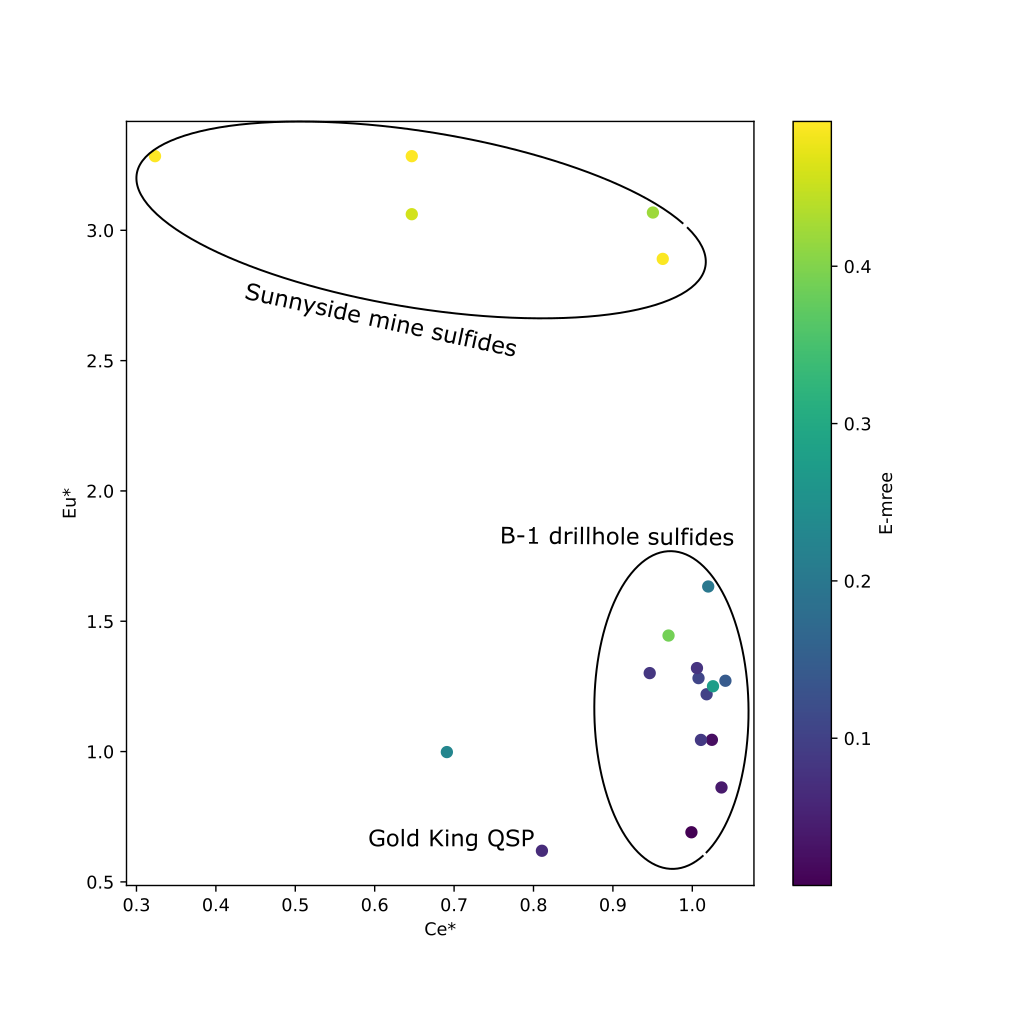


**Fig. S5.** Plot of Ce* versus Eu* colored by E_MREE_ for solid samples from Casedevall and Ohmoto (1977).


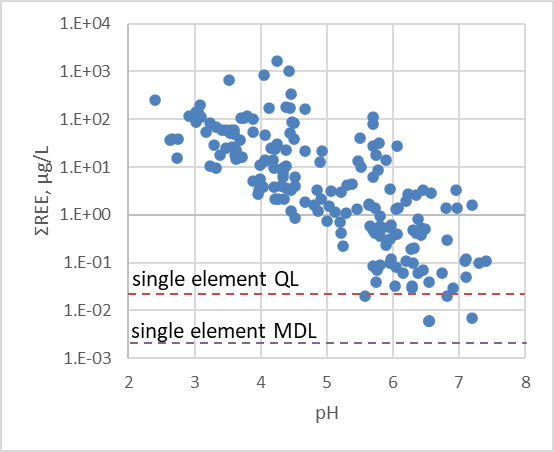


**Fig. S6.** Plot of total REE versus pH in samples collected during 2018 -2020. QL = quantitation limit; MDL = method detection limit.

**Table S3.** Values of Ce/Ce* and Eu/Eu* from all samples. * indicates anomalies wherein one of the three elements used in the calculation is affected by censored values (less than QL or MDL).

| **Site ID** | **Sample Date** | **Site Type** | **Ce/Ce*** | **Eu/Eu*** |
| --- | --- | --- | --- | --- |
| ATPZ-02 | 09/14/2020 | Groundwater well | 0.66 | 2.84* |
| CC01B | 09/25/2018 | Mine | 0.88 | 1.29 |
| CC01B | 09/17/2019 | Mine | 0.88 | 1.24 |
| CC03C | 10/01/2018 | Mine | 0.79 | 0.98 |
| CC03C | 09/18/2019 | Mine | 0.81 | 1.03 |
| CC06 | 10/09/2018 | Mine | 0.98 | 1.45 |
| CC06 | 09/19/2019 | Mine | 0.95 | 1.29 |
| CC14 | 10/09/2018 | Mine | 0.87 | 0.98 |
| CC14 | 09/18/2019 | Mine | 0.82 | 0.98 |
| CC19 | 10/09/2018 | Mine | 0.90 | 0.86 |
| CC50 | 10/03/2018 | Mine | 0.68 | 0.86 |
| CC50 | 09/18/2019 | Mine | 0.73 | 1.15 |
| NATW572 | 08/17/1998 | Mine | 0.98 | 1.07 |
| NATW622 | 08/21/1998 | Mine | 0.71 | 2.33 |
| NAW135 | 08/13/1997 | Mine | 0.64 | 1.94 |
| NAW144 | 08/13/1997 | Mine | 0.23 | 1.90 |
| NAW150 | 08/13/1997 | Mine | 0.93 | 1.03 |
| NAW156 | 08/14/1997 | Mine | 0.98 | 1.26 |
| NAW161 | 08/14/1997 | Mine | 0.92 | 1.54 |
| NAW176 | 08/15/1997 | Mine | 0.75 | 1.22 |
| NAW192 | 08/16/1997 | Mine | 0.92 | 1.46 |
| NAW201 | 08/16/1997 | Mine | 0.93 | 1.15 |
| NAW204 | 08/17/1997 | Mine | 0.92 | 1.26 |
| NAW212 | 08/17/1997 | Mine | 0.87 | 1.12 |
| NAW213 | 08/17/1997 | Mine | 1.00 | 1.20 |
| NAW215 | 08/17/1997 | Mine | 0.90 | 1.63 |
| NAW217 | 08/17/1997 | Mine | 0.99 | 1.37 |
| NAW226 | 08/17/1997 | Mine | 0.88 | 0.96 |
| NAW227 | 08/17/1997 | Mine | 0.90 | 0.97 |
| NAW231 | 08/18/1997 | Mine | 0.83 | 1.20 |
| NAW232 | 08/18/1997 | Mine | 0.80 | 1.18 |
| NAW235 | 08/18/1997 | Mine | 0.77 | 1.57 |
| NAW271 | 08/19/1997 | Mine | 0.23 | 2.83 |
| NAW298 | 08/21/1997 | Mine | 0.73 | 4.82 |
| NAW309 | 08/21/1997 | Mine | 0.97 | 1.22 |
| NAW311 | 08/21/1997 | Mine | 0.97 | 1.36 |
| NAW329 | 09/04/1997 | Mine | 0.95 | 1.19 |
| NAW355 | 09/05/1997 | Mine | 0.28 | 4.95 |
| NAW364 | 09/06/1997 | Mine | 0.49 | 4.82 |
| NAW396 | 09/08/1997 | Mine | 0.36 | 4.82 |
| NAW399 | 09/09/1997 | Mine | 0.42 | 3.48 |
| NAW412 | 09/11/1997 | Mine | 0.94 | 1.07 |
| NAW415 | 09/11/1997 | Mine | 0.78 | 1.05 |
| NAW427 | 09/11/1997 | Mine | 0.86 | 1.10 |
| NAW508 | 08/13/1998 | Mine | 0.93 | 1.70 |
| NAW512 | 08/14/1998 | Mine | 0.88 | 1.44 |
| NAW516 | 08/14/1998 | Mine | 0.92 | 1.59 |
| NAW517 | 08/14/1998 | Mine | 0.95 | 1.56 |
| NAW520 | 08/14/1998 | Mine | 0.99 | 1.22 |
| NAW521 | 08/14/1998 | Mine | 0.94 | 1.18 |
| NAW524 | 08/14/1998 | Mine | 1.24 | 1.36 |
| NAW530 | 08/15/1998 | Mine | 0.90 | 1.35 |
| NAW551 | 08/16/1998 | Mine | 0.87 | 1.50 |
| NAW552 | 08/16/1998 | Mine | 0.98 | 0.99 |
| NAW554 | 08/16/1998 | Mine | 0.97 | 0.97 |
| NAW555 | 08/17/1998 | Mine | 1.03 | 1.19 |
| NAW570 | 08/17/1998 | Mine | 0.99 | 1.03 |
| NAW574 | 08/17/1998 | Mine | 0.42 | 2.84 |
| NAW575 | 08/17/1998 | Mine | 1.77 | 2.33 |
| NAW579 | 08/18/1998 | Mine | 0.20 | 0.25 |
| NAW580 | 08/18/1998 | Mine | 0.93 | 1.15 |
| NAW582 | 08/18/1998 | Mine | 0.20 | 0.45 |
| NAW588 | 08/18/1998 | Mine | 1.04 | 0.85 |
| NAW589 | 08/18/1998 | Mine | 1.04 | 1.21 |
| NAW592 | 08/19/1998 | Mine | 0.88 | 0.06 |
| NAW598 | 08/19/1998 | Mine | 0.86 | 0.95 |
| NAW601 | 08/19/1998 | Mine | 1.77 | 1.85 |
| NAW603 | 08/20/1998 | Mine | 0.52 | 2.41 |
| NAW626 | 08/21/1998 | Mine | 0.09 | 3.28 |
| NAW630 | 08/22/1998 | Mine | 1.00 | 1.17 |
| NAW647 | 08/23/1998 | Mine | 0.20 | 2.41 |
| NAW648 | 08/23/1998 | Mine | 0.45 | 6.57 |
| NAW649 | 08/23/1998 | Mine | 0.20 | 3.28 |
| NAW651 | 08/23/1998 | Mine | 0.20 | 4.01 |
| NAW704 | 09/11/1998 | Mine | 0.74 | 1.56 |
| NAW707 | 09/12/1998 | Mine | 0.20 | 3.28 |
| NAW713 | 09/12/1998 | Mine | 1.05 | 0.85 |
| NAW717 | 09/13/1998 | Mine | 0.89 | 1.06 |
| NAW721 | 09/13/1998 | Mine | 0.45 | 1.67 |
| NAW723 | 09/13/1998 | Mine | 0.20 | 3.28 |
| NAW725 | 09/13/1998 | Mine | 0.92 | 1.68 |
| NAW726 | 09/14/1998 | Mine | 1.03 | 1.57 |
| NAW727 | 09/14/1998 | Mine | 0.91 | 1.27 |
| NAW733 | 09/14/1998 | Mine | 0.95 | 1.43 |
| NAW735 | 09/15/1998 | Mine | 0.39 | 3.28 |
| NAW741 | 09/16/1998 | Mine | 0.12 | 3.00 |
| SS046 | 09/25/2018 | Mine | 0.96 | 1.07 |
| SS046 | 09/17/2019 | Mine | 0.96 | 1.10 |
| SS105 | 09/17/2019 | Mine | 0.85 | 0.98 |
| SS110 | 10/01/2018 | Mine | 0.56 | 1.35 |
| SS111 | 10/01/2018 | Mine | 0.91 | 1.01 |
| SS112 | 10/01/2018 | Mine | 0.42 | 3.80 |
| SS112 | 09/19/2019 | Mine | 0.31 | 2.41 |
| SS115 | 10/01/2018 | Mine | 0.82 | 0.86 |
| SS001 | 09/26/2018 | Seeps/Springs | 0.36 | 1.15 |
| SS004 | 09/26/2018 | Seeps/Springs | 0.02 | 0.37 |
| SS006 | 09/26/2018 | Seeps/Springs | 0.10 | 4.82* |
| SS007 | 09/26/2018 | Seeps/Springs | 0.88 | 1.18 |
| SS010 | 09/24/2018 | Seeps/Springs | 0.14 | 0.95 |
| SS011 | 09/24/2018 | Seeps/Springs | 0.58 | 1.17 |
| SS013 | 10/04/2018 | Seeps/Springs | 0.68 | 1.47 |
| SS015 | 10/04/2018 | Seeps/Springs | 0.67 | 2.78* |
| SS016 | 10/04/2018 | Seeps/Springs | 0.86 | 1.07 |
| SS017 | 10/04/2018 | Seeps/Springs | 0.92 | 1.09 |
| SS018 | 10/04/2018 | Seeps/Springs | 0.95 | 1.03 |
| SS019 | 10/04/2018 | Seeps/Springs | 0.27 | 2.41 |
| SS020 | 09/25/2018 | Seeps/Springs | 0.44 | 0.87 |
| SS023 | 09/25/2018 | Seeps/Springs | 0.28 | 1.90 |
| SS024 | 09/25/2018 | Seeps/Springs | 0.02 | 3.21* |
| SS027 | 09/24/2018 | Seeps/Springs | 0.32 | 1.21 |
| SS029 | 09/24/2018 | Seeps/Springs | 0.07 | 1.06 |
| SS029 | 09/19/2019 | Seeps/Springs | 0.06 | 1.15 |
| SS030 | 09/26/2018 | Seeps/Springs | 0.06 | 0.96 |
| SS032 | 09/26/2018 | Seeps/Springs | 0.04 | 1.54* |
| SS032 | 09/19/2019 | Seeps/Springs | 0.94 | 1.45* |
| SS060 | 10/09/2018 | Seeps/Springs | 0.33 | 0.90 |
| SS060 | 09/16/2019 | Seeps/Springs | 0.33 | 0.97 |
| SS062 | 10/08/2018 | Seeps/Springs | 0.78 | 0.94 |
| SS062 | 09/18/2019 | Seeps/Springs | 0.82 | 0.95 |
| SS067 | 10/08/2018 | Seeps/Springs | 0.95 | 0.95 |
| SS067 | 09/19/2019 | Seeps/Springs | 0.93 | 1.00 |
| SS069 | 10/09/2018 | Seeps/Springs | 0.87 | 0.88 |
| SS069 | 09/18/2019 | Seeps/Springs | 0.12 | 0.99 |
| SS069 | 09/20/2019 | Seeps/Springs | 0.84 | 0.90 |
| SS084 | 09/19/2019 | Seeps/Springs | 0.98 | 1.33 |
| SS086 | 10/09/2018 | Seeps/Springs | 0.96 | 0.97 |
| SS126 | 10/03/2018 | Seeps/Springs | 0.98 | 1.36 |
| SS200 | 10/08/2018 | Seeps/Springs | 0.52 | 4.66 |
| SS200 | 09/19/2019 | Seeps/Springs | 0.53 | 2.68 |
| SS201 | 10/04/2018 | Seeps/Springs | 1.06 | 0.82 |
| SS208 | 10/03/2018 | Seeps/Springs | 0.88 | 0.21 |
| SS219 | 10/01/2018 | Seeps/Springs | 0.73 | 1.48 |
| SS220 | 10/01/2018 | Seeps/Springs | 0.66 | 0.36 |
| SS236 | 10/08/2018 | Seeps/Springs | 0.95 | 1.02 |
| SS236 | 09/20/2019 | Seeps/Springs | 0.93 | 1.11 |
| SS250 | 10/09/2018 | Seeps/Springs | 0.71 | 3.80* |
| SS250 | 09/19/2019 | Seeps/Springs | 0.53 | 1.57* |
| SS300 | 10/09/2018 | Seeps/Springs | 0.22 | 0.95 |
| SS301 | 10/04/2018 | Seeps/Springs | 1.20 | 0.79 |
| SS301 | 09/23/2019 | Seeps/Springs | 1.12 | 0.86 |
| NAW507 | 08/13/1998 | Stream | 0.91 | 1.22 |
| NAW510 | 08/13/1998 | Stream | 0.84 | 1.32 |
| NAW511 | 08/14/1998 | Stream | 0.86 | 0.79 |
| NAW518 | 08/14/1998 | Stream | 0.93 | 1.13 |
| NAW538 | 08/15/1998 | Stream | 0.99 | 1.35 |
| NAW539 | 08/16/1998 | Stream | 0.90 | 1.11 |
| NAW543 | 08/16/1998 | Stream | 0.96 | 1.38 |
| NAW549 | 08/16/1998 | Stream | 0.90 | 0.91 |
| NAW562 | 08/17/1998 | Stream | 1.02 | 1.06 |
| NAW568 | 08/17/1998 | Stream | 1.03 | 1.11 |
| NAW581 | 08/18/1998 | Stream | 0.93 | 1.21 |
| NAW610 | 08/20/1998 | Stream | 0.20 | 4.01 |
| NAW611 | 08/20/1998 | Stream | 0.99 | 3.28 |
| NAW632 | 08/22/1998 | Stream | 0.29 | 0.54 |
| NAW634 | 08/22/1998 | Stream | 0.20 | 1.04 |
| NAW635 | 08/22/1998 | Stream | 0.88 | 1.23 |
| NAW644 | 08/22/1998 | Stream | 0.70 | 2.33 |
| NAW683 | 09/10/1998 | Stream | 0.92 | 1.24 |
| NAW719 | 09/13/1998 | Stream | 0.24 | 2.98 |
| NAW729 | 09/14/1998 | Stream | 0.68 | 1.08 |
| A72 | 09/15/2020 | Stream | 0.80 | 1.04 |
| CC48 | 09/15/2020 | Stream | 0.85 | 1.09 |
| M34 | 09/15/2020 | Stream | 0.84 | 1.19 |

# References

Bowen, G.J., 2024, Online isotopes in precipitation calculator, version 3.1, available at https://wateriso.utah.edu/waterisotopes/pages/data_access/oipc.html, accessed 4/8/2024.

Casadevall, T. and Ohmoto, H., 1977 Sunnyside Mine, Eureka mining district, San Juan County, Colorado; geochemistry of gold and base metal ore deposition in a volcanic environment: Economic Geology, v. 72, no. 7, p. 1285–1320, DOI: 10.2113/gsecongeo.72.7.1285.

Newman, C.P., Runkel, R.L., Cowie, R., and Wilkin, R.T., 2024, Compilation of water-quality data, discharge data, and geochemical equilibrium models for streams, draining mine adits, and springs in the Upper Animas River Watershed, 1987 – 2020: U.S. Geological Survey data release, DOI: 10.5066/P9OOHY1Q.

U.S. Environmental Protection Agency, 1994a, Method 200.7: Determination of Metals and Trace Elements in Water and Wastes by Inductively Coupled Plasma-Atomic Emission Spectrometry, https://www.epa.gov/esam/method-2007-determination-metals-and-trace-elements-water-and-wastes-inductively-coupled.

U.S. Environmental Protection Agency, 1994a, EPA Method 200.8: Determination of Trace Elements in Waters and Wastes by Inductively Coupled Plasma-Mass Spectrometry, https://www.epa.gov/esam/epa-method-2008-determination-trace-elements-waters-and-wastes-inductively-coupled-plasma-mass.

Wilkin, R.T., Lee, T.R., Ludwig, R.D., Wadler, C., Brandon, W., Mueller, B., Davis, E., Luce, D., and Edwards, T., 2021, Rare-earth elements as natural tracers for in-situ remediation of groundwater: Environmental Science and Technology, v. 55, p. 1251-1259, DOI: 10.1021/acs.est.0c06113.

Yager, D.B. and Bove, D.J., 2007, Geologic framework, pp. 111-137, in: Church, S.E., von Guerard, P., and Finger, S.E., eds., Integrated investigation of environmental effects of historical mining in the Animas River Watershed, San Juan County, Colorado: U.S. Geological Survey Professional Paper 1651, pp. 1096 plus CD-ROM.
